# *ENAM* mutations and digenic inheritance

**DOI:** 10.1002/mgg3.928

**Published:** 2019-09-02

**Authors:** Hong Zhang, Yuanyuan Hu, Figen Seymen, Mine Koruyucu, Yelda Kasimoglu, Shih‐Kai Wang, John Timothy Wright, Michael W. Havel, Chuhua Zhang, Jung‐Wook Kim, James P. Simmer, Jan C.‐C. Hu

**Affiliations:** ^1^ Department of Biologic and Materials Sciences University of Michigan School of Dentistry Ann Arbor MI USA; ^2^ Department of Pedodontics, Faculty of Dentistry Istanbul University Istanbul Turkey; ^3^ Department of Dentistry National Taiwan University School of Dentistry Taipei City Taiwan R.O.C.; ^4^ Department of Pediatric Dentistry University of North Carolina School of Dentistry Chapel Hill NC USA; ^5^ Department of Molecular Genetics and Department of Pediatric Dentistry and Dental Research Institute, School of Dentistry Seoul National University Seoul Korea

**Keywords:** amelogenesis imperfecta, enamel, hypoplasia, tooth

## Abstract

**Background:**

*ENAM* mutations cause autosomal dominant or recessive amelogenesis imperfecta (AI) and show a dose effect: enamel malformations are more severe or only penetrant when both *ENAM* alleles are defective.

**Methods:**

Whole exome sequences of recruited AI probands were initially screened for mutations in known AI candidate genes. Sanger sequencing was used to confirm sequence variations and their segregation with the disease phenotype. The co‐occurrence of *ENAM* and *LAMA3* mutations in one family raised the possibility of digenic inheritance. Enamel formed in *Enam^+/+^Ambn* ^+/+^, *Enam^+/−^*, *Ambn^+/−^*, and *Enam^+/−^Ambn^+/−^* mice was characterized by dissection and backscattered scanning electron microscopy (bSEM).

**Results:**

*ENAM* mutations segregating with AI in five families were identified. Two novel *ENAM* frameshift mutations were identified. A single‐nucleotide duplication (c.395dupA/p.Pro133Alafs*13) replaced amino acids 133‐1142 with a 12 amino acid (ATTKAAFEAAIT*) sequence, and a single‐nucleotide deletion (c.2763delT/p.Asp921Glufs*32) replaced amino acids 921‐1142 with 31 amino acids (ESSPQQASYQAKETAQRRGKAKTLLEMMCPR*). Three families were heterozygous for a previously reported single‐nucleotide *ENAM* deletion (c.588+1delG/p.Asn197Ilefs*81). One of these families also harbored a heterozygous *LAMA3* mutation (c.1559G>A/p.Cys520Tyr) that cosegregated with both the AI phenotype and the *ENAM* mutation. In mice, *Ambn^+/−^* maxillary incisors were normal. *Ambn^+/−^* molars were also normal, except for minor surface roughness. *Ambn^+/−^* mandibular incisors were sometimes chalky and showed minor chipping. *Enam^+/−^* incisor enamel was thinner than normal with ectopic mineral deposited laterally. *Enam^+/−^* molars were sometimes chalky and rough surfaced. *Enam^+/−^Ambn^+/−^* enamel was thin and rough, in part due to ectopic mineralization, but also underwent accelerated attrition.

**Conclusion:**

Novel *ENAM* mutations causing AI were identified, raising to 22 the number of *ENAM* variations known to cause AI. The severity of the enamel phenotype in *Enam^+/−^Ambn^+/−^* double heterozygous mice is caused by composite digenic effects. Digenic inheritance should be explored as a cause of AI in humans.

## 1 INTRODUCTION

The most fundamental feature of dental enamel formation is the deposition of thin, parallel, evenly spaced mineral ribbons of amorphous calcium phosphate on freshly mineralized dentin collagen fibers and elongation of those ribbons along a mineralization front in close proximity to the secretory surface of the ameloblast distal membrane (Simmer, Richardson, Hu, Smith, & Hu, 2012). The mineral ribbons extend in the direction that the ameloblast distal membrane retreats from the dentin surface (Boyde, 1967; Smith, Hu, Hu, & Simmer, 2016). Enamel ribbon formation first evolved in fish and still forms today in gar (a ray‐finned fish with gills and a vascularized swim bladder that could serve as lungs) (Sire, 1994), in Coelacanth (a lobe‐finned fish with lungs) (Kawasaki & Amemiya, 2014), lungfish (Satchell, Shuler, & Diekwisch, 2000), and tetrapods, including humans (Ronnholm, 1962). The common ancestor for gar and tetrapods diverged about 450 million years ago, so amelogenesis evolved before then (Braasch et al., 2016; Kawasaki et al., 2017). The genes that encode the secreted proteins necessary for early enamel formation include enamelin (*ENAM*, OMIM *606585) (Hu et al., 1997), ameloblastin (*AMBN*, *601259) (Krebsbach et al., 1996), amelogenin (*AMELX*, OMIM *300391) (Snead et al., 1985), and matrix metallopeptidase 20 (*MMP20*, *604629) (Bartlett, Simmer, Xue, Margolis, & Moreno, 1996). Secreted proteins are critical for early amelogenesis, and little or no true enamel forms in *Enam* (Hu et al.,^2014^, ^2008^; Hu, Lertlam, et al., 2011; Seedorf et al., 2007; Smith et al., 2016, 2009), *Ambn* (Fukumoto et al., 2004; Wazen, Moffatt, Zalzal, Yamada, & Nanci, 2009), *Amelx* (Gibson et al., 2001; Smith et al., 2016), or *Mmp20* (Bartlett, Beniash, Lee, & Smith, 2004; Caterina et al., 2002; Hu et al., 2016) null mice. These genes are specialized for dental enamel formation and become pseudogenized in vertebrates that stop making teeth or dental enamel during evolution (Meredith, Gatesy, Cheng, & Springer, 2011; Meredith, Gatesy, Murphy, Ryder, & Springer, 2009; Meredith, Gatesy, & Springer, 2013; Springer et al., 2016). Because these genes are enamel specific and function early (during the secretory stage of amelogenesis when the enamel ribbons elongate to establish the final thickness of the enamel layer), humans with inherited malformations caused by defects in these genes often exhibit hypoplastic (thin) forms of isolated amelogenesis imperfecta (AI).

Enamelin is particularly critical for dental enamel formation (Hu & Yamakoshi, 2003). When both *Enam* alleles are defective or absent in mice, no true enamel layer forms (Smith et al., 2016). As the dentin mineral continues to thicken, no enamel ribbons are deposited on the dentin surface, and ameloblasts become increasingly pathological, and some epithelial cells undergo apoptosis (Hu, Lertlam, et al., 2011). Instead of enamel, ameloblasts in *Enam* ^‐/‐^ mice deposit a thin, disorganized mineralized crust (rather than true enamel) on the dentin surface as well as ectopic calcifications within the enamel organ (Hu et al., 2014; Smith et al., 2009). Enamelin function is dependent upon phosphorylation by Golgi Casein Kinase (Cui et al., 2015). The absence of a single phosphorylation causes severe enamel defects in humans (Chan et al., 2010) and in mice (Yan et al., 2017).

The enamel phenotypes caused by *ENAM* mutations in humans show a clear dose effect. When a single *ENAM* allele is defective, the enamel malformations vary in severity and can be nonpenetrant (Seymen et al., 2014). The mildest observable enamel defects are well‐circumscribed enamel pits, often arranged in horizontal lines (Hart, Hart, et al., 2003), or horizontal grooves, usually in the cervical third of the crown (Kang et al., 2009; Mardh et al., 2002), with the most severe heterozygous phenotype being profound generalized thinning of the enamel layer (Hart, Michalec, Seow, Hart, & Wright, 2003; Kida, Ariga, Shirakawa, Oguchi, & Sakiyama, 2002). When both *ENAM* alleles are defective, the enamel layer is either completely absent or appears as a very thin mineral layer that only partially covers the crown (Hart, Hart, et al., 2003; Ozdemir et al., 2005; Simmer, Estrella, Milkovich, & Hu, 2013). The severity of enamel defects caused by a single defective *Enam* allele in mice varies in different teeth. *Enam^+/−^* maxillary incisors deposit virtually normal enamel, while the enamel mineral formed on *Enam^+/−^* mandibular incisors is reduced by as much as 50% (Smith et al., 2009).

Nearly 20 genes have been found to cause isolated (nonsyndromic) forms of AI, and more AI‐causing genes have yet to be identified (Smith et al., 2017). Global efforts are underway to identify the genes and mutations that cause inherited enamel defects. By identifying the critical participants in amelogenesis, genetic studies are improving our understanding of the basic mechanisms of dental enamel formation while providing the means for genetic testing to diagnose the cause of inherited enamel malformations in patients. Many genes are also known to cause AI in syndromes (Wright, Carrion, & Morris, 2015), where the enamel malformations often precede the onset of other phenotypes, including kidney calcifications (Jaureguiberry et al., 2012; Wang, Aref, et al., 2013), distal renal tubular acidosis (Rungroj et al., 2018; Zhang et al., 2019), proximal renal tubular acidosis (Demirci, Chang, Mah, Romero, & Gorin, 2006), immunodeficiency (Lacruz & Feske, 2015), and blindness (Parry et al., 2009). A diagnosis that identifies the genetic cause gives the affected family the relief of knowing their condition is isolated, or awareness that future health risks go along with their enamel malformations and might be mitigated by therapeutic interventions.

Candidate gene approaches for genetic testing to identify the etiology in AI cases are impractical due to the increasing number of AI candidate genes and unnecessary because of advances in high‐throughput DNA sequencing, which can identify sequence variations throughout the exome in a single experiment (Wang, Hu, et al., 2013). Whole exome sequence (WES) analyses can also identify potential digenic inheritance, the simplest form of genetically complex diseases (Schaffer, 2013). Although human enamel malformations are not yet associated with digenic inheritance, *Mmp20^+/−^*/*Klk4^+/−^* double heterozygous mice show an enamel phenotype, whereas the single heterozygous (*Mmp20^+/−^* or *Klk4^+/−^*) mice do not (Hu et al., 2016).

In this study we identify five families with hypoplastic AI caused by dominant heterozygous or recessive compound heterozygous *ENAM* mutations. All six affected members of one family also expressed a potentially deleterious heterozygous *LAMA3* defect, raising the possibility of digenic effects. The enamel phenotypes of *Enam^+/−^* and *Ambn^+/−^* mice were compared with *Enam^+/−^*/*Ambn^+/−^* double heterozygous mice, demonstrating composite digenic effects in mice harboring a single allele *Enam* defect.

## 2 MATERIALS AND METHODS

### 2.1 Recruitment and ethical compliance

The study protocol and subject consent forms were reviewed and approved by the Ethics Committee at the University of Istanbul, and the Institution Review Boards at the University of Michigan, the University of North Carolina, and National Taiwan University Hospital. Two unrelated Turkish families with generalized hypoplastic enamel were characterized and recruited by Dr. Seymen and her team. Two unrelated Caucasian families with hypoplastic AI were recruited by Dr. Tim Wright at the University of North Carolina and Dr. Jan Hu at the University of Michigan School of Dentistry. A Taiwanese family was recruited by Dr. Shih‐Kai Wang at National Taiwan University. Study explanation, pedigree construction, subject enrollment, clinical examinations, and collection of blood or saliva samples were completed under the proper consenting procedure specified in the study protocols and according to the Declaration of Helsinki.

This study complied with US National Research Council's Guide for the Care and Use of Laboratory Animals, the US Public Health Service's Policy on Humane Care and Use of Laboratory Animals, and Guide for the Care and Use of Laboratory Animals.

### 2.2 Whole‐exome sequencing and bioinformatics analysis

Either the nonstimulated saliva sample of 2 ml or peripheral blood sample of 5 ml was collected from each participant. Each sample was inspected, coded, then a small aliquot was removed for genomic DNA isolation following the manufacturer's protocol. Genomic DNA quality was assessed by 1.5% agarose gel electrophoresis and quantity was determined using a Qubit^TM^ Fluorometer (ThermoFisher Scientific). Samples from the parents and proband of each family were selected for whole‐exome sequencing (WES), while DNA samples from the other family members were used for segregation analyses. DNA samples, following the initial quality control, were submitted to Johns Hopkins Center for Inherited Disease Research (CIDR) for WES. Each DNA sample, at the concentration of 50 ng/µl, volume of 50 µl, and total amount of 2.5 µg, was plated onto a 96‐well plate. A manifest file with coded sample information and the plated samples were shipped to the CIDR overnight on dry ice. Each sample was genotyped using an Illumina QC Array. Once sample aliquoting errors were ruled out, and the performance potential and genotypes were determined to be appropriate, then samples were subjected to the WES procedure. Exome capture was completed using the SureSelect Human All Exon Enrichment System (Agilent Technologies). Paired‐end sequencing was generated using the Illumina HiSeq 2500 (CIDR). Sequencing reads were aligned to the 1000 genomes phase 2 (GRCh37) human genome reference using BWA version 0.7.8 (Li & Durbin, 2010). Duplicate reads were flagged with Picard version 1.109. Local realignment around indels and base call quality score recalibration was performed using the Genome Analysis Toolkit (GATK) (McKenna et al., 2010) version v3.3‐0. GATK’s reference confidence model workflow was used to perform joint sample genotyping to generate a multi‐sample VCF file. Variant filtering was done using the Variant Quality Score Recalibration (VQSR) method (DePristo et al., 2011). Multi‐sample VCF files from each family containing variants that were polymorphic among the family members were extracted from the multi‐sample VCF file derived from the specific cohort with similar phenotypes. All variants in individual VCF files were annotated using VarSeq (Golden Helix) against a variety of data sources including gene annotation, function prediction, and frequency information (a cutoff value of 0.01 for the minor allele frequency). Following the comparisons between the affected and unaffected individuals, a list of prioritized variants was then subjected to segregation analysis. *ENAM* mutations are reported with respect to the first nucleotide of the *ENAM* genomic reference sequence (NG_013024.1) and the first nucleotide of the *ENAM* translation initiation site in the *ENAM* cDNA reference (NM_031889.2). The *LAMA3* sequence variation associated with the disease phenotype in Family 5 is reported with respect to the first nucleotide of the translation initiation site in the *LAMA3* cDNA reference sequence (NM_198129.2).

### 2.3 Segregation analyses using sanger sequencing

The prioritized DNA sequence variations and their segregation within each family was confirmed by Sanger sequencing. The PCR primers were designed to bracket the candidate variant, and the reactions were conducted following the established protocols (Kim et al., 2005). PCR primers and amplification conditions are provided in Table S1.

### 2.4 Dissection microscopy

*Enam^+/−^* and *Ambn^+/−^* mice (C57BL/6 background) were crossed to generate *Enam* ^+/+^ *Ambn* ^+/+^ (WT), *Enam^+/−^Ambn^+/−^* (double heterozygous), *Enam^+/−^*, and *Ambn^+/−^* (single heterozygous) mice. Seven‐week‐old mice were anesthetized with isofluorane, sacrificed, displayed on a Nikon SMZ1000 dissection microscope equipped with a Nikon digital camera DXM1200 (Melville, NY) and photographed. The mandibles were removed and sliced through the mental symphysis with a razor blade. The hemimandibles were dissected free of soft tissues using tissue forceps and a spoon excavator, submerged in 1% NaClO for 5 min, rinsed, air dried, and photographed.

### 2.5 Scanning electron microscopy

Tissue processing: 7‐week‐old mice were anaesthetized with isoflurane and fixed by cardiac perfusion. Blood was cleared from the vasculature using lactated Ringer's solution (30–45 s) followed by 4% paraformaldehyde in PBS (135 mM NaCl, 2.7 mM KCl, 4.3 mM Na_2_HPO_4_, 1.4 mM Na_2_H_2_PO_4_; pH 7.3) for 20 min. The mouse mandibles were removed and stripped of soft tissue, fixed by immersion in 4% paraformaldehyde overnight, and washed in PBS 3x (every 0.5–1 hr) with one overnight wash at 4°C. The embedding, cross‐sectioning, polishing and imaging using a Cameca SX‐100 Electron Microprobe Analyzer on backscatter mode were described previously (Hu, Hu, Smith, Bartlett, & Simmer, 2011).

## 3 RESULTS

Whole exome sequence analyses of genomic DNA obtained from families with amelogenesis imperfecta (AI) identified five probands with potential disease‐causing mutations in *ENAM*. Sanger sequencing confirmed the presence of these *ENAM* mutations, determined their distribution among the recruited members in each family, and established segregation of the *ENAM* defects with the AI phenotype.

### 3.1 Family 1

**Figure 1:**
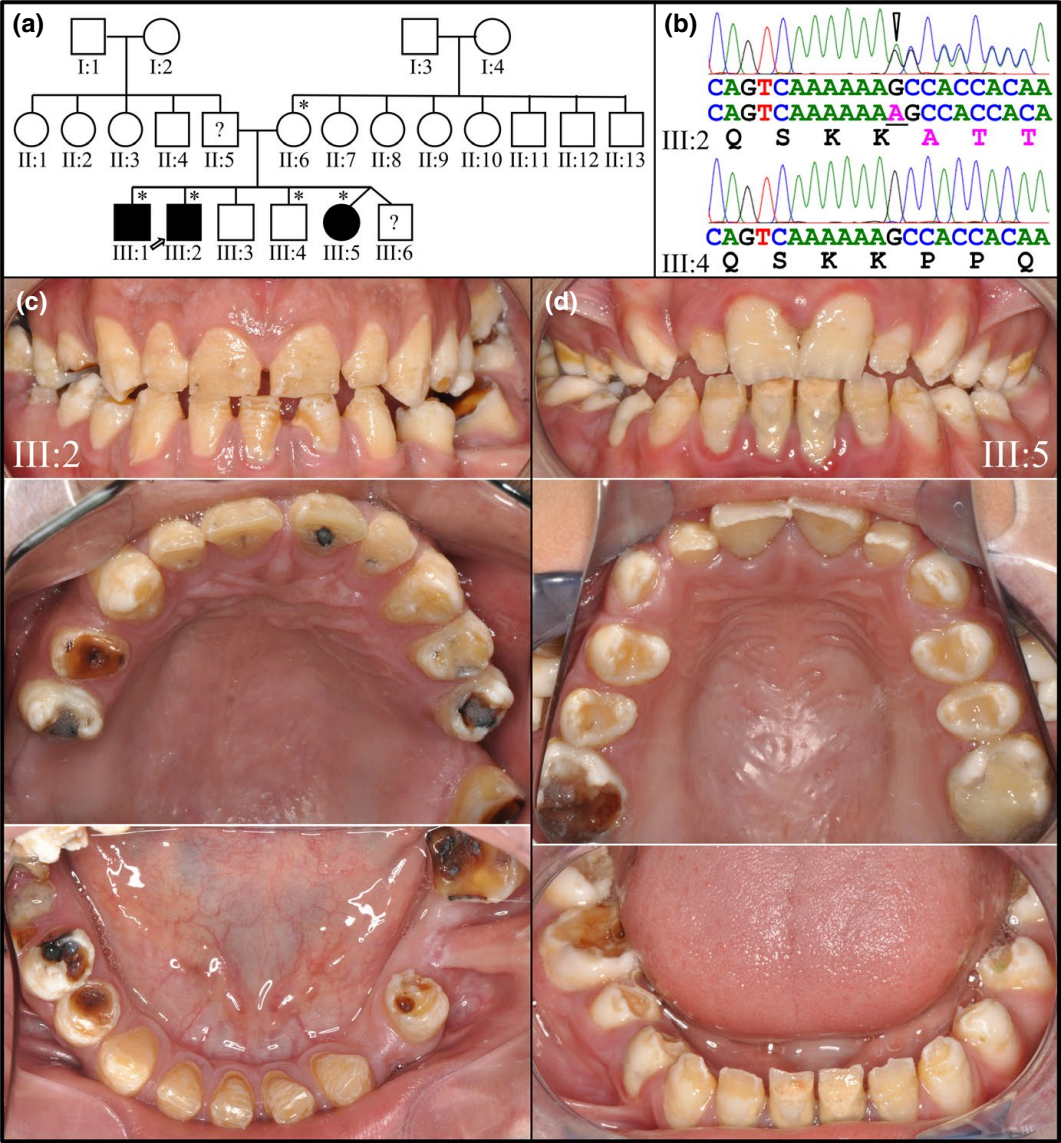
Family 1. (a) Pedigree of Family 1. Asterisks mark the five recruited members, three of which were affected. The proband (III:2) is indicated by an arrow. (b) DNA sequence chromatograms confirmed the WES results showing that the proband (III:2) was heterozygous for the *ENAM* mutation NM_031889.2: c.395dupA/p.(Pro133Alafs*13), as were his affected older brother (III:1) and younger sister (III:5). The DNA sequence chromatogram for the younger brother (III:4) and mother (II:6) matched the reference sequence, and these individuals had normal enamel (Figure S2). (c) Clinical photos of the proband at age 24 showing generalized hypoplastic enamel with horizontal hypoplastic grooves on the mandibular anterior teeth as well as several large occlusal surface carious lesions. (d) Clinical photos of the proband's affected younger sister (III:5) at age 13, also showing generalized hypoplastic enamel

The enamel malformations in Family 1 (Figure 1) followed an autosomal dominant pattern of inheritance with no consanguinity. Five members of this Turkish family were recruited, with three being affected (Figure 1a). The three affected members harbored one defective *ENAM* allele that had a frameshift mutation in Exon 7 (XM_006714056.4: c.395dupA/p.Pro133Alafs*13) (Figure 1b), which has not been reported previously (Table S2). This single‐nucleotide duplication truncates the enamelin protein (normally 1,142 amino acids) after Lys132 and replaces amino acids 133–1142 with the 12 amino acid sequence ATTKAAFEAAIT. The aberrant transcript from the mutant allele may not be translated into protein as its mRNA is likely to undergo nonsense mediated decay (Kurosaki & Maquat, 2016). The three affected individuals (III:1, III:2, and III:5) were all heterozygous for this frameshift mutation, while the two unaffected individuals (II:6 and III:4) did not have this mutation on either *ENAM* allele and had normal enamel (Figure S1). The proband's father (II:5) and oldest brother were not enrolled and their affection statuses and genotypes are unknown, although it is presumed that the three affected siblings characterized in this study inherited the mutant *ENAM* allele from their father. The proband (III:2) presented with generalized hypoplastic enamel with brown discoloration, pitting, and marked attrition of the occlusal and incisal surfaces (Figure 1c). The anterior teeth exhibited horizontal grooves and banding, predominantly on their buccal surfaces. The incisal edges of these teeth (at age 24) showed signs of wear and chipping on their incisal edges. The posterior dentition was affected by dental caries, with large cavitated carious lesions mainly on occlusal surfaces. The bicuspids had rough surfaces where the enamel had apparently crumbled near the cusp tips. The proband's affected younger sister at age 13 (III:5; Figure 1d) displayed similar hypoplastic enamel with generalized interdental spacing, anterior incisal wear, chipping and horizontal banding, and posterior chipping of cusps, occlusal wear, and occlusal caries. The younger sister's (III:5) teeth were not as brown as those of the proband. The oldest brother (III:1) was affected, but the enamel phenotype was relatively mild (Figure S2). His teeth were not significantly stained (Figure S3a). The panorex detected generalized enamel hypoplasia (Figure S2c), although interdental spacing was only observed between the anterior teeth. Many well‐circumscribed enamel pits were observed most notably on buccal surfaces. The more severe enamel phenotypes of the younger siblings (III:2 and III:5) relative to their mildly affected older brother (III:1) with the same heterozygous *ENAM* defect suggests sequence variations in other genes might contribute to the severity of the enamel defects.

### 3.2 Family 2

We performed whole‐exome sequence analyses of genomic DNA from two affected and two unaffected members of a nonconsanguineous Caucasian family, referred to us with a diagnosis of autosomal recessive amelogenesis imperfecta. Two different *ENAM* mutations were identified (Figure S3). The first was a frameshift mutation in Exon 10 (NM_031889.2: c.1259_1260insAG/p.Pro422Valfs*27) that had previously been reported to cause AI (Chan et al., 2010; Hart, Hart, et al., 2003; Kang et al., 2009; Lindemeyer, Gibson, & Wright, 2010; Ozdemir et al., 2005; Pavlic, Petelin, & Battelino, 2007; Wright et al., 2011). This mutation (rs587776588) causes a frameshift after Gly421 that deletes amino acids 422–1142 and replaces them with 26 extraneous amino acids VPNLALLFAMKKSKIQRRSPWVQKNK*. The defect was observed in one *ENAM* allele (heterozygous) in the unaffected father (II:9) and in his two affected daughters (III:1 and III:2). The second *ENAM* defect (NM_031889.2: c.2763delT/p.Asp921Glufs*32) was also a frameshift mutation in Exon 10. This novel frameshift truncated the enamelin protein after Arg920 and replaced amino acids 921–1142 with 31 extraneous amino acids ESSPQQASYQAKETAQRRGKAKTLLEMMCPR*. This frameshift was identified in the unaffected mother (II:8) and in her two affected daughters (III:1 and III:2). Therefore, the unaffected parents were simple heterozygotes for one of the frameshifts, while both of the affected children were complex heterozygotes with frameshifts in both *ENAM* alleles (Figure S3). The novel frameshift mutation (p.Asp921Glufs*32) has not been previously reported to cause amelogenesis imperfecta, but had been submitted to the dbSNP database without any clinical information (rs529979202). These two frameshift mutations in Family 2 are in Exon 10, the most 3′ *ENAM* exon, making them more likely to escape nonsense mediated decay than premature stop codons in upstream exons (Kurosaki & Maquat, 2016).

### 3.3 Family 3

**Figure 2:**
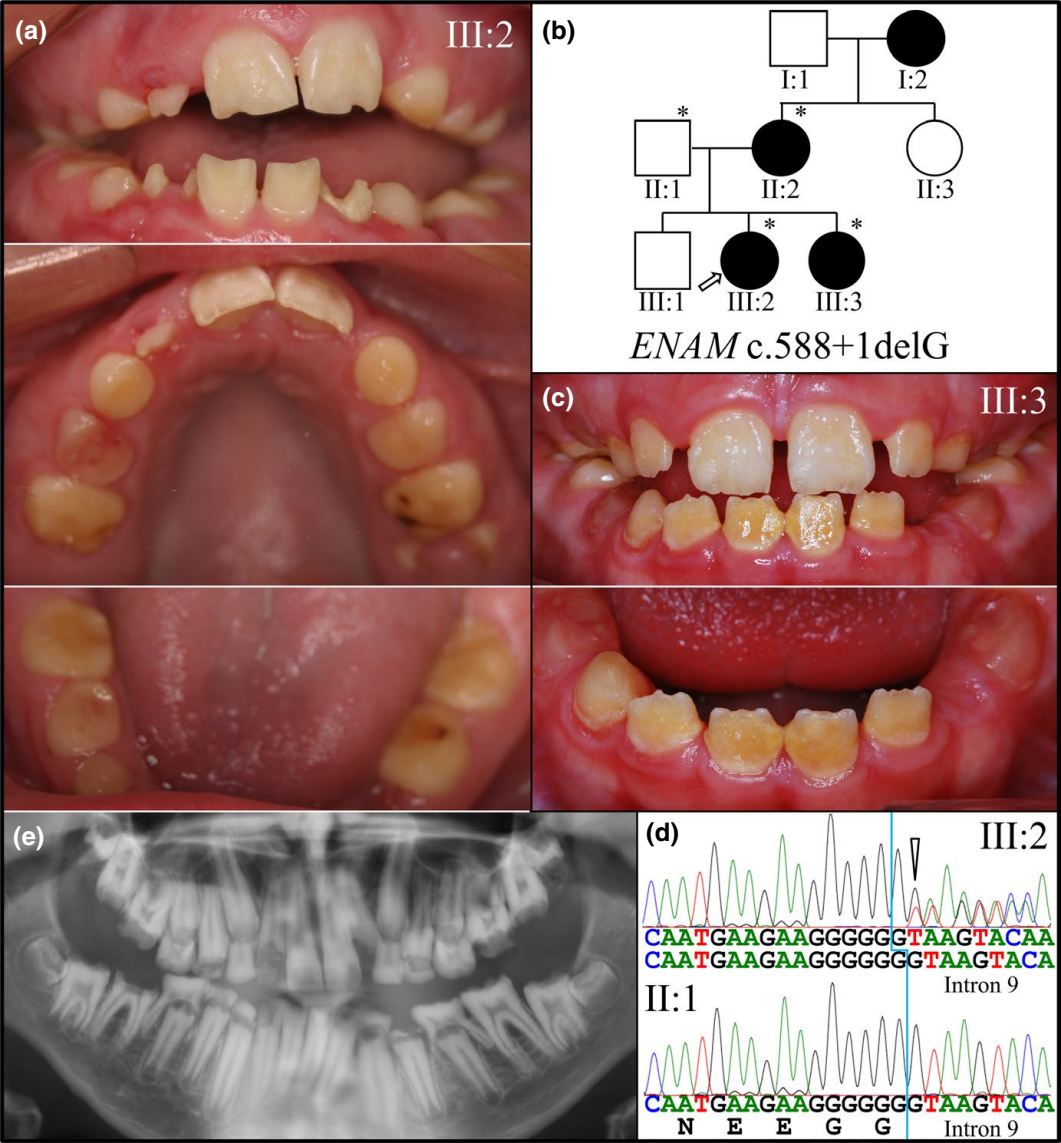
Family 3. (a) Clinical photos of the proband (III:2) at age 12 in the mixed dentition stage of dental development and exhibiting severely hypoplastic enamel, interdental spacing, premature attrition, and chipping of the more recently erupted permanent incisors. (b) Pedigree showing autosomal dominant inheritance. (c) Clinical photos of the affected younger sister (III:3) at age 10 also showing severely hypoplastic enamel, interdental spacing, premature attrition, and chipping of the incisors. (d) The three recruited affected members of the family were heterozygous for the *ENAM* c.588+1delG/(p.Asn197Ilefs*81) mutation in Exon 9/Intron 9 causing a frameshift in the mRNA. The three affected members were heterozygous for this *ENAM* frameshift while the unaffected father (II:1) lacked the mutation. (e) Panoramic radiograph of the proband (III:2) showing mixed dentition, with little or no enamel evident

In Family 3, four members of a nonconsanguineous Caucasian family were recruited, three of whom were affected (Figure 2). All affected family members (II:2, III:2, and III:3), but not the unaffected father (II:1), had a heterozygous *ENAM* c.588+1delG mutation. The deleted guanine (G) follows a run of six Gs at the end of *ENAM* Exon 9 (Figure 2d). Functionally, the sixth G (the seventh being deleted) acts as the first nucleotide of the splicing donor site (GT), so splicing occurs normally, but Exon 9 ends up with one less nucleotide (G), causing a frameshift (p.Asn197Ilefs*81) that truncates the enamelin protein after 197 amino acids (which normally has 1,142 amino acids), and adds 80 extraneous amino acids to the C‐terminus of the truncated protein (Table S2). Deletion of any one of the seven consecutive Gs results in this frameshift. The *ENAM* c.588+1delG mutation causes a severe, readily detected ADAI enamel phenotype and is the most frequently reported *ENAM* defect (Hart, Hart, et al., 2003; Kida et al., 2002; Kim et al., 2005; Pavlic, Battelino, Trebusak Podkrajsek, & Ovsenik, 2011; Pavlic et al., 2007; Wright et al., 2011). Although reported to be a mutational “hot spot,” the mutation is rare, and is not listed in the 1000 Genomes database (Ensembl GRCh38 of the 1000 Genomes Project data) (1000 Genomes Project Consortium et al., 2015) or National Center for Biotechnology Information (NCBI) Single Nucleotide Polymorphism Database (dbSNP) (Kitts & Sherry, 2012), which includes short deletion and insertion polymorphisms (indels/DIPs) as well as single‐nucleotide polymorphisms (SNPs). A similar *ENAM* defect in which a G in the same run of seven Gs was duplicated rather than deleted (rs1085307975; NG_013024.1: c.588+1dupG; p.Asn197Glufs*25) has been identified by clinical testing and submitted to the dbSNP, but the enamel phenotype was not described or published.

Although only one *ENAM* allele is defective, the enamel is markedly reduced in thickness, and on many crowns the enamel appears to be nonexistent. Generalized, severely hypoplastic enamel has been observed in all reported cases of AI caused by heterozygous *ENAM* c.588+1delG mutations. In previous cases where the primary teeth were available for scanning electron microscopic (*SEM*) analysis, the enamel was thin and lacked prism organization (Hart, Hart, et al., 2003; Pavlic et al., 2007). This same *ENAM* mutation was identified in Families 4 and 5.

### 3.4 Family 4

**Figure 3:**
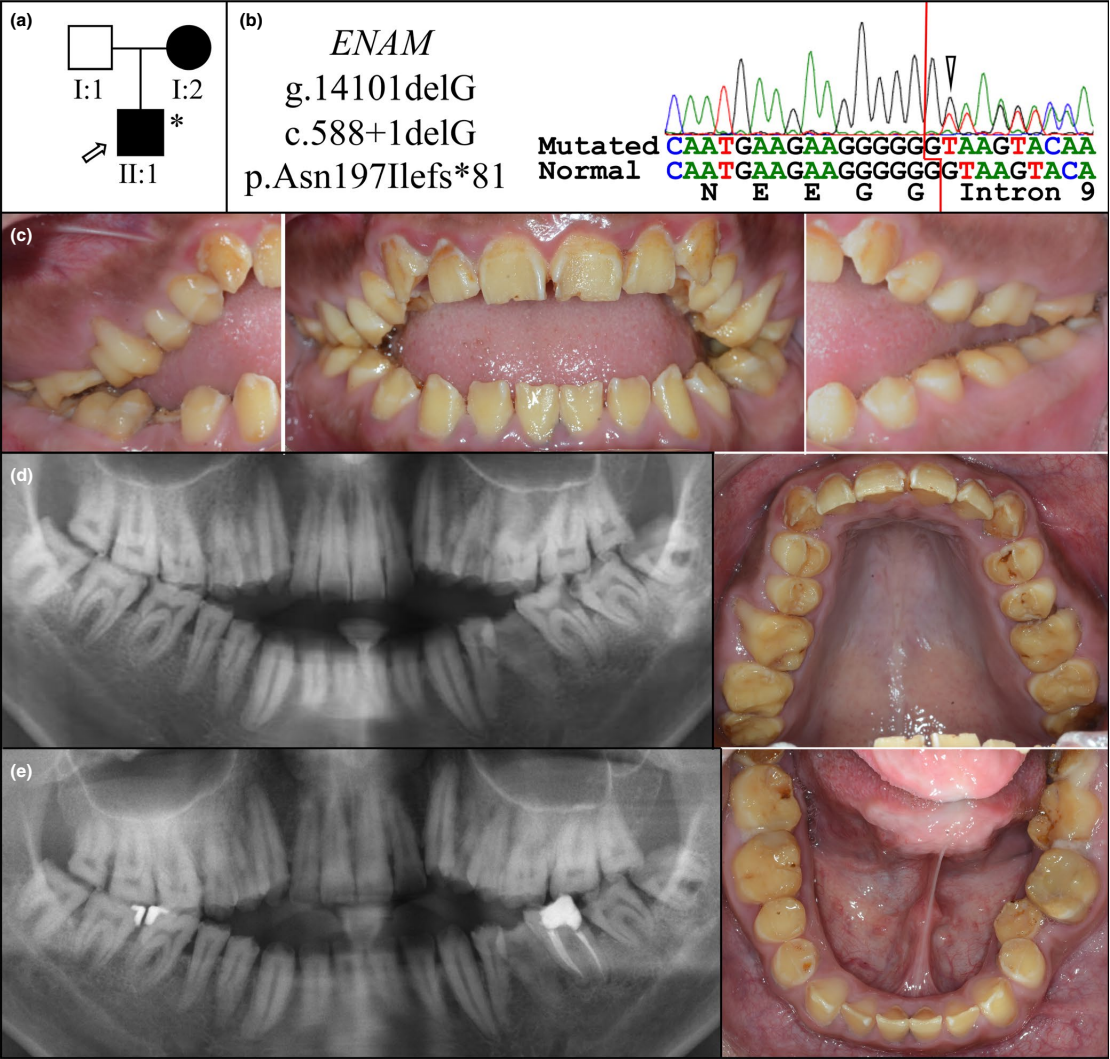
Family 4. (a) Pedigree showing autosomal dominant inheritance. (b) The proband was heterozygous for the *ENAM* c.588+1delG/ p.(Asn197Ilefs*81) frameshift mutation in Exon 9/Intron 9. (c) Clinical photos of the proband (II:1) at age 25 exhibiting severely hypoplastic enamel, interdental spacing, and premature attrition and an anterior open bite. (d) Panoramic radiograph of the proband (II:1) at age 22 showing little or no enamel evident. (e) Panoramic radiograph of the proband (II:1) at age 25 following restoration of carious lesions

Family 4 was a Taiwanese nuclear family of presumed East Asian descent (Figure 3). The proband was a 25‐year‐old male presenting with generalized severe enamel hypoplasia but was otherwise healthy. Clinically, there was almost no enamel on all the permanent teeth, except some thin patches found at the proximal area of upper incisors and lower anterior teeth, corresponding to the findings on panoramic radiographs. On posterior teeth, there was also a thin band‐like enamel structure around the middle of the tooth crown. Narrowed dental arch forms with a high palatal vault and anterior open bite were also evident. Exome analysis of proband's DNA identified the recurrent *ENAM* c.588+1delG mutation without any other potential disease‐causing mutation in known AI candidate genes. The *ENAM* mutation was confirmed by Sanger sequencing.

### 3.5 Family 5 (Digenic Effects?)

**Figure 4:**
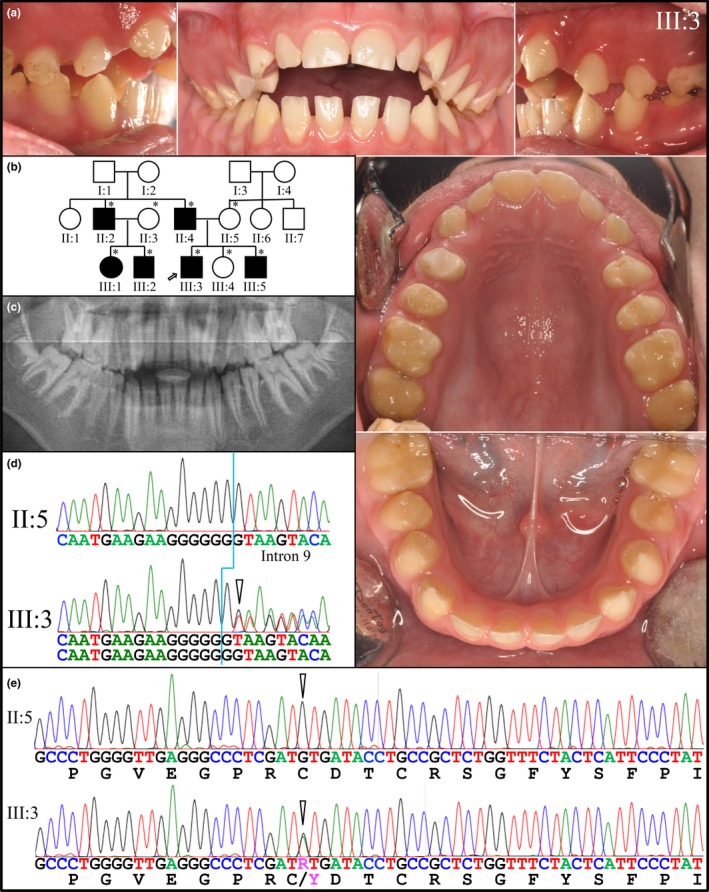
Family 5. (a) Clinical photos of the proband (III:3) at age 14 showing generalized thin, hypoplastic enamel with pitting on the buccal surfaces of the maxillary premolars and an anterior open bite. (b) Pedigree of Family 4 showing an apparent autosomal dominant pattern of inheritance. Asterisks indicate family members that were recruited for this study, nine in total. The proband is marked by an arrow. (c) Panoramic image of the proband. Very little enamel is evident, either because it is absent or does not contrast with dentin (is hypomineralized). Two potential disease‐causing mutations were identified: (d)* ENAM*: c.588+1delG/ p.Asn197Ilefs*81, which was previously reported to cause ADAI in multiple families (Hart, Hart, et al., 2003; Kang et al., 2009; Kida et al., 2002; Pavlic et al., 2007; Wright et al., 2011). (e)* LAMA3* missense mutation c.1559G>A/p.Cys520Tyr. All recruited affected family members (II:2, II:4, III:1, III:2, III:3, and III:5) were heterozygous for both of these (*ENAM* and *LAMA3*) mutations. All recruited unaffected family members (II:3, II:5, and III:4) had neither mutation. No family members were heterozygous for the *ENAM* or *LAMA3* mutation alone

In Family 5, the proband at age 14 (III:3) presented with generalized hypoplastic AI, interdental spacing, and an anterior open bite (Figure 4). The maxillary right premolars exhibited localized pitting and brown staining on the buccal surfaces that was not observed on other teeth (Figure 4a). There was evidence of enamel attrition, such as large wear facets and chipped enamel. The incisal edges of the mandibular anteriors and premolars had fractured or chipped, plausibly due to the interplay of malformed enamel and misaligned occlusion (Figure 4a). Nine members of this nonconsanguineous Turkish family were recruited, six of which were affected (Figure 4b). The enamel malformations followed an autosomal dominant pattern of inheritance (Figure 4b). There was little or no evidence of enamel on the panoramic radiograph. If enamel was present, it did not contrast well with dentin and could not be distinguished from it (Figure 4c). All six affected family members, and none of the three unaffected persons, had a heterozygous *ENAM* c.588 +1delG/p.Asn197Ilefs*81 mutation as well as a heterozygous *LAMA3* c.1559G>A/p.Cys520Tyr mutation. The enamel phenotypes of the unaffected mother (II:3) and affected father (II:4) are provided in Figure S4. Those of the two affected cousins, age 11 and 9 (III:1 and III:2) are shown in Figure S5, and the enamel phenotypes of the unaffected younger sister, age 11 (III:4) and affected younger brother, age 8 (III:5), are displayed in Figure S6. Despite sharing the same *ENAM* mutation, Family 3 and Family 4 are of different ethnicity, so the *ENAM* mutation almost certainly occurred independently in the two families.

The *LAMA3* missense mutation is rare, is not listed in dbSNP, and is predicted to interfere with protein function (Sorting Intolerant from Tolerant (SIFT) score = 0 (Sim et al., 2012); Polyphen2 score = 1.0 (Adzhubei, Jordan, & Sunyaev, 2013). Unfortunately, these two unlinked genes (*ENAM* Chr. 4q13.3; *LAMA3* Chr. 18q11.2) cosegregated in all of the six recruited affected individuals and were both absent from all three of the recruited, unaffected members of the family, so the phenotypic consequence of the heterozygous *LAMA3* defects alone could not be observed. Other heterozygous *LAMA3* mutations are reported to cause localized enamel hypoplasia and pitting (Gostynska et al., 2016; Yuen, Pasmooij, Stellingsma, & Jonkman, 2012), but the effects on enamel of this particular heterozygous *LAMA3* mutation (NM_198129.2: c.1559G>A/p.Cys520Tyr) have not been reported. In Family 4, the *ENAM* c.588+1delG mutation caused a severe generalized enamel hypoplasia and localized pitting, so the potential contribution of the *LAMA3* defect to the enamel phenotype in this family is unclear. The low frequency of the defect and the prediction software results indicate that the heterozygous *LAMA3* c.1559G>A/p.Cys520Tyr mutation would likely cause AI, but such evidence is not decisive by itself (Miosge et al., 2015), so the enamel pathogenicity of this *LAMA3* defect remains uncertain.

### 3.6 Mouse *Enam*‐*Ambn* digenic effects

**Figure 5:**
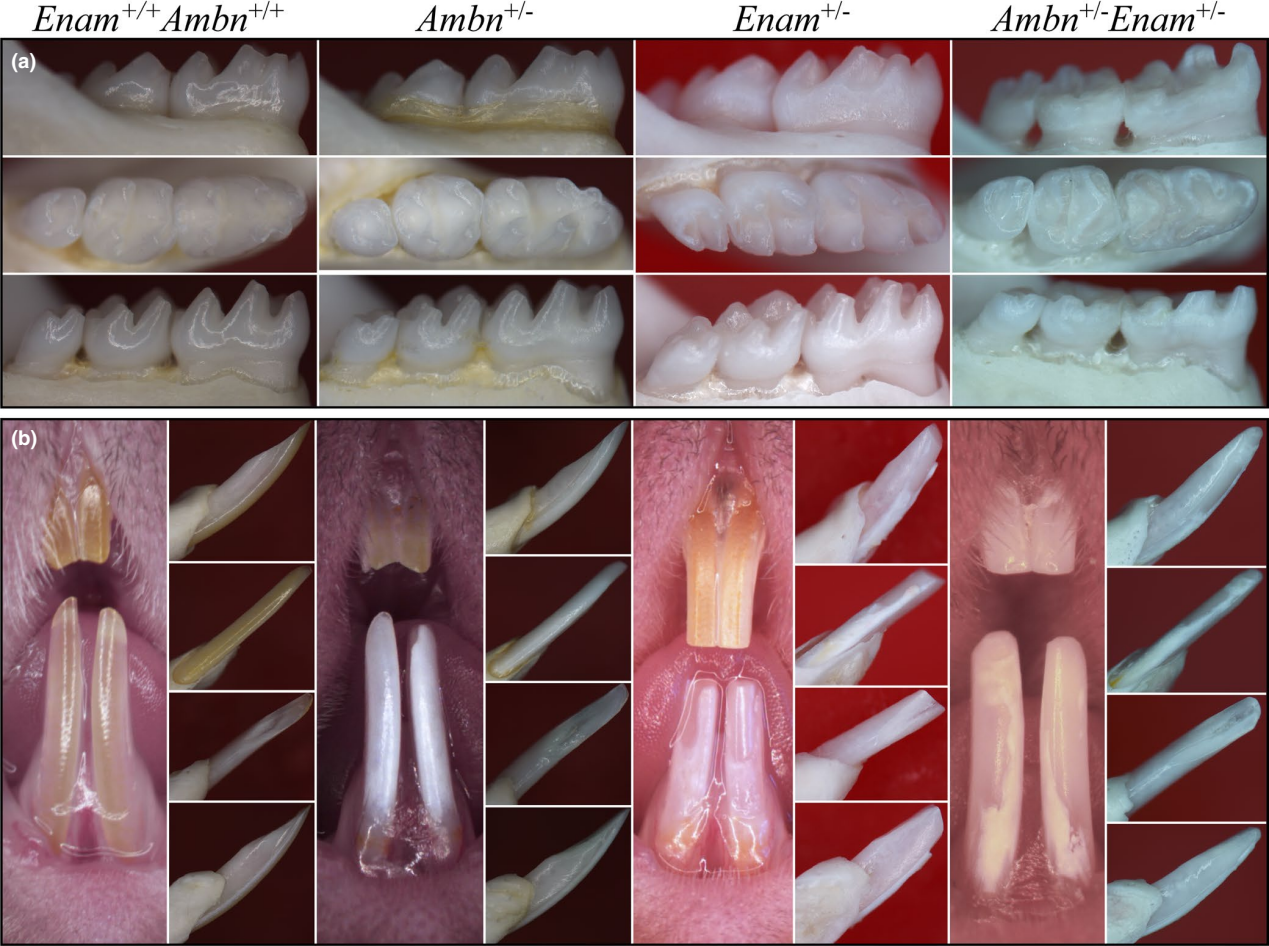
Dissecting light microscopy comparison of 7‐week *Ambn* ^+/+^ *Enam* ^+/+^, *Ambn^+/−^*, *Enam^+/−^*, and *Ambn^+/−^Enam^+/−^* molars and incisors. (a) Buccal, occlusal and lingual views of mandibular molars. The *Ambn^+/−^* and *Enam^+/−^* molars closely resembled the wild type in crown form and level of attrition. The *Ambn^+/−^* molars showed minor surface roughness. The *Enam^+/−^* molars showed increased surface roughness and a chalky appearance. The *Ambn^+/−^Enam^+/−^* molars showed clear digenic effects on their enamel: more severe surface roughness, chalkiness, and rapid attrition than either single heterozygote. (b) Mesial, buccal, lingual, and lateral views of the mandibular incisors and facial view of the maxillary and mandibular incisors. Maxillary incisor chalkiness and mandibular incisor attrition both follow the pattern of progressively increasing severity going from *Ambn^+/−^* to *Enam^+/−^* to *Ambn^+/−^Enam^+/−^* mice, indicative of digenic effects

To help assess the potential for digenic effects to contribute to the enamel phenotype in *ENAM* heterozygotes, *Enam* ^‐/‐^ (Hu et al., 2008) and *Ambn* ^‐/‐^ (Liang et al., 2019) mice were crossed to generate double heterozygous offspring. The erupted dental phenotypes of 7‐week‐old wild‐type, single heterozygous, and double heterozygous mice were first evaluated by dissection microscopy. The *Ambn^+/−^* maxillary incisors and molars both resembled the wild type (Figure 5a). The *Ambn^+/−^* mandibular incisors were chalky white and sometimes showed minor chipping (Figure 5b). The *Enam^+/−^* molars closely resembled the wild type in overall shape, but the enamel surface was rough textured (Figure 5b). The *Enam^+/−^Ambn^+/−^* molars showed more severe surface roughness, chalkiness, and greater attrition than either of single heterozygous mice. The *Enam^+/−^* mandibular incisor enamel was chalky white and consistently chipped. The *Enam^+/−^Ambn^+/−^* double heterozygous maxillary and mandibular incisors were both chalky white and much of the mandibular incisor enamel had been lost to attrition. Even at low magnification under a dissecting microscope digenic effects on surface roughness, chalkiness, and rate of attrition were evident on both molar and incisors.

**Figure 6:**
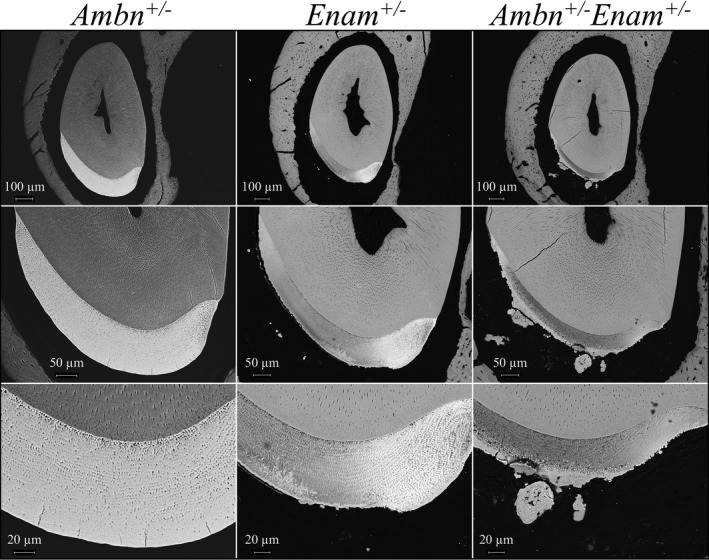
Level 8 cross‐sections from 9‐week *Ambn^+/−^*, *Enam^+/−^*, and *Ambn^+/−^Enam^+/−^* mouse mandibular incisors imaged by backscattered scanning electron microscopy (bSEM). The *Ambn^+/−^* incisor is similar to the wild type. The *Enam^+/−^* incisors exhibit a somewhat thinner enamel with normal‐looking enamel rods. The enamel is rough, with some ectopic mineral nodules on its surface. The *Ambn^+/−^Enam^+/−^* incisors shows a more severe enamel phenotype, with thin enamel and large ectopic nodules that appeared to form as concretions. Severe enamel malformations were consistently found in the *Ambn^+/−^Enam^+/−^* mouse (Figure S7)

Mandibular incisors at 7 weeks were cross‐sectioned at the level of the labial alveolar crest (prior to this portion of the incisor erupting into the oral cavity) and characterized by bSEM (Figure 6 and Figure S7). The enamel layer looked nearly normal in the *Ambn^+/−^* mandibular incisors, with characteristic rows of enamel rods (Smith, Hu, Hu, & Simmer, 2019). The *Enam^+/−^* mandibular incisor enamel showed normal rod patterns, but the enamel was thinner and part of the outer enamel layer did not form properly and its surface was rough. *Enam^+/−^Ambn^+/−^* mandibular incisor was thinner still, had a rough, crusty surface, and ectopic concretions in the soft tissue.

**Figure 7:**
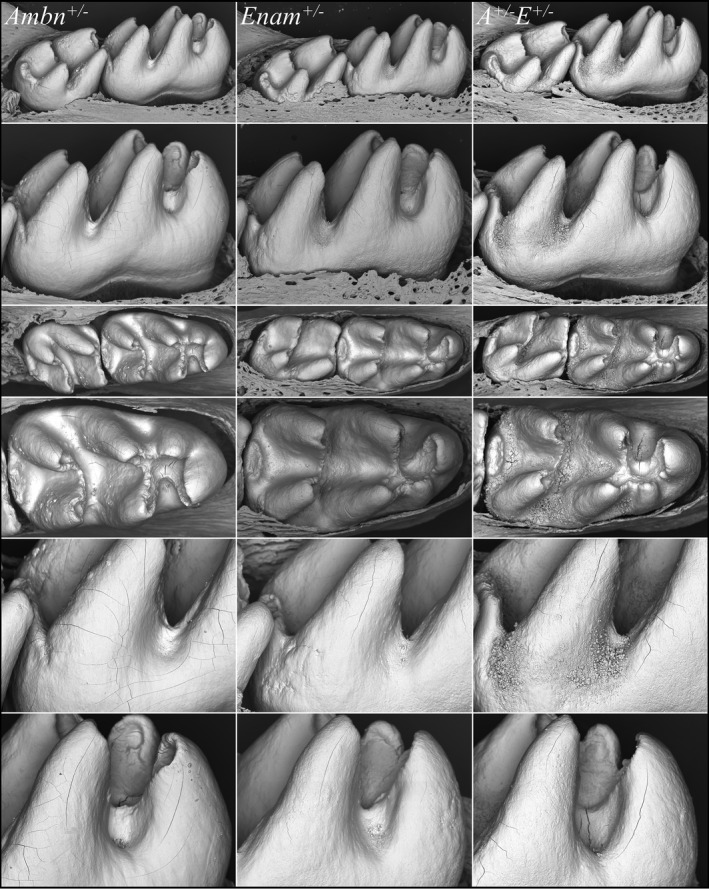
bSEM images of D14 mouse molars from *Ambn^+/−^*, *Enam* ^‐/‐^, and *Ambn^+/−^Enam^+/−^* mice. These images were taken after removing the soft tissue covering these erupting molars. The *Ambn^+/−^* (Figure S8) and *Enam* ^‐/‐^ (Figure S9) molars resembled the wild type except for showing localized areas of surface roughness. The *Ambn^+/−^Enam^+/−^* mouse molars showed extensive areas of surface roughness and regions covered by a mineral crust. A crusty, rough surface was a consistent finding in *Enam^+/−^Ambn^+/−^* mice (Figure S10)

To assess the form and surface textures of the single and double heterozygous mouse molars after they had fully formed but before they could be altered in the oral cavity, we removed the overlying soft tissue from nearly erupted D14 mandibular first molars and examined them using bSEM (Figure 7). The overall shape of the *Ambn^+/−^* (Figure S8) and *Enam^+/−^* (Figure S9) D14 mandibular first molar crowns was normal, with minor surface roughness. In contrast, the D14 mandibular first molars showed major surface roughness with surface nodules (Figure S10). The enamel defects observed in incisors and molars were consistently most severe in the *Enam^+/−^Ambn^+/−^* double heterozygotes relative to the *Ambn^+/−^* or *Enam^+/−^* single heterozygous mice.

## 4 DISCUSSION

Enamelin was expressed when enamel first formed on the scales and teeth of fish more than 450 million years ago. Enamelin is specifically expressed by secretory stage ameloblasts and its primary structure has been conserved during evolution (Gasse & Sire, 2015). Analysis of 36 known mammalian *Enam* sequences identified 77 unchanged and 346 conservative amino acid positions distributed throughout the eight coding exons with conserved intron boundaries, demonstrating significant selection pressure to maintain *ENAM* structure and function (Al‐Hashimi, Sire, & Delgado, 2009). Defects in *ENAM* also cause inherited enamel defects in dogs. A five base pair deletion in *Enam* (c.1991_1995delTTTCC/p.Phe665Argfs*3) causes nonsyndromic recessive AI in Italian Greyhounds, which manifests as rough, thin enamel with brownish mottling (Gandolfi, Liu, Griffioen, & Pedersen, 2013).

This report increases to 22 the number of reported novel human *ENAM* mutations that cause AI, which are widely distributed across the coding exons and intron borders (Table S2). *ENAM* disease‐causing mutations include small deletions and insertions that truncate the protein, shift the reading frame, and add potentially toxic C‐terminal adducts. There are also splice donor and acceptor site, missense, and stop‐gain mutations. The enamel phenotypes caused by *ENAM* mutations sometimes show incomplete penetrance and variable expressivity. *ENAM* heterozygotes sometimes display no detectable AI phenotype (lack of penetrance) (Seymen et al., 2014), but might still affect the enamel in subtle ways. A combination of two *ENAM* amino acid substitutions (p.Ile648Thr and p.Arg763Gln) expressed from the same allele (which did not cause AI) were associated with a two‐ to threefold increase in risk for caries susceptibility (Chaussain et al., 2014).

Including Families 3, 4, and 5 from this study, the *ENAM* c.588+1delG mutation has been identified in seven unrelated families (Table S2) with various ethnicities, suggesting that this is a mutation “hot spot.” The mutation‐prone nature of this spot might be attributed to its mononucleotide repeat sequence of seven Gs (Figure 3c). Tandemly repeated sequences have long been recognized as hot spots for frameshift mutations, presumably caused by “DNA polymerase slippage” (Strauss, 1999). This mechanism involves a transient dissociation of the replication DNA template and a re‐annealing within the repeat tract, forming an extrahelical loop comprised of one or more repeat units, which would in turn cause an insertion or deletion. A characteristic of the repeated sequences in humans is the large contribution of runs of As in noncoding regions of the genome, which is more permissive. In contrast, repeat runs in coding regions are restricted by the amino acid code, and a run of glycines (GGG) might not be tolerated (Strauss, 1999). In *E. coli*, repeats of Gs and Cs were observed to frameshift more frequently than repeats of As in a mismatch repair deficient strain (Sagher, Hsu, & Strauss, 1999). The *ENAM* c.588+1delG mutation, removing one G out of seven, should allow normal splicing between Exon 9 and Intron 9, but cause a frameshift (p.Asn197Ilefs*81) that introduces a premature termination codon in the last exon (Exon 10). This mutant transcript would presumably escape nonsense mediated decay and produce a mutant ENAM protein. This mutant protein, carrying 80 aberrant amino acids at its C‐terminus, might interact with the wild‐type ENAM protein or other EMPs, causing a dominant negative effect. Alternatively, the mutant protein might be toxic to ameloblasts and cause cell pathology, which leads to a more severe enamel defect when compared with phenotypes caused by simple loss‐of‐function mutations in a single *ENAM* allele. The *ENAM* c.588+1delG enamel defect is readily recognizable clinically and hard to overlook, which might also contribute to the frequent identification of this mutation.

*ENAM* mutations show a dose effect: when only one allele is defective the phenotype is nonpenetrant, or milder than when both alleles are defective. The enamelin dose effect is largely due to the fact that detectable enamel malformations are often observed when only one *ENAM* allele is defective (dominant pattern of inheritance). Most genes that are required for amelogenesis are expressed at sufficiently high levels that a loss of one allele (haploinsufficiency) does not cause a detectable enamel phenotype and produces a recessive pattern of inheritance. Over‐expression as well as under‐expression of early secreted enamel proteins interferes with enamel ribbon deposition. Expression of *Ambn* (Teepe et al., 2014) or *Mmp20* (Shin, Chavez, Ikeda, Foster, & Bartlett, 2018) transgenes in wild‐type mice already expressing these genes at normal levels causes enamel malformations. It is plausible then that natural selection limits *ENAM* expression levels to less than twice the amount required to avoid over‐expression defects, resulting in an autosomal dominant pattern of inheritance with a dose effect. In any case, amelogenesis is a sensitive process that can be readily disturbed. Here, we establish that digenic effects can cause AI in mice.

The Online Mendelian Inheritance in Man (OMIM) lists 91 conditions having an enamel phenotype, with 71 having a known genetic etiology (Wright et al., 2015). About half of AI conditions show a recessive pattern of inheritance (Wright et al., 2011). A genetic search for the etiology of AI in a given family has a success rate of about a 60% (Wright et al., 2011). A disproportionate share of the unsolved cases is recessive. There are multiple reasons why cases go unsolved, but the most likely is that there are still many genes that cause AI that have not yet been identified. Another reason is the inherent difficulty in determining the etiology when more than one gene is involved. Inherited enamel malformations are highly heterogeneous and some are likely to be caused by variations in gene regulatory sequences and digenic inheritance.

## CONFLICT OF INTEREST

The authors have no conflict of interest.
