# Supplementary material for: ENAM mutations and digenic inheritance
Source: Mol Genet Genomic Med. 2019 Sep 2;7(10):e00928. doi: 10.1002/mgg3.928 (PMC6785452; doi:10.1002/mgg3.928)
Supplement: Supplementary file 1 [file MGG3-7-e00928-s001.pdf]

Family 1. *ENAM* Exon 7: NG_013024.1:g.10749dupA; NM_031889.2:c.395dupA; p.(Pro133Alafs*13)
Primers generate a 641 bp amplification product.
7F: TGCCTTTTGGTTTGTTTTGG
7R: AGTCGACTTGCCATGCAGTT; RC: AACTGCATGGCAAGTCGACT
PCR conditions: template denaturation @ 94 °C for 2 min, then [35 cycles of 94 °C for 15 s (primer annealing) then 59 °C for 30s (primer extension)] followed by 68 °C for 50 s, 68 °C for 2 min and then hold at 4 °C.

Family 2. *ENAM* Exon 10: NG_013024.1:g.18942_18943insAG; NM_031889.2:c.1259_1260insAG; p.(Pro422Valfs*27)
10FB: CAGGAAACAGTACCCCAGGA
10RB: GGGAAGGATGGGGTAAATGT; RC: ACATTTACCCCATCCTTCCC
Primers generate a 966 bp amplification product.
PCR conditions: template denaturation @ 94 °C for 2 min, then [35 cycles of 94 °C for 15 s (primer annealing) then 59 °C for 30 s (primer extension)] followed by 68 °C for 60 s, 68 °C for 2 min and then hold at 4 °C.

Family 2. *ENAM* Exon 10: NG_013024.1:g.20446delT; NM_031889.2:c.2763delT; p.(Asp921Glufs*32)
10FE: CCCTAACTTCATCCCACCAA
10RE: TCATGGCCTTCTTGCTTTTT; RC: AAAAAGCAAGAAGGCCATGA
Primers generate a 1128 bp amplification product.
PCR conditions: template denaturation @ 94 °C for 2 min, then [35 cycles of 94 °C for 15 s (primer annealing) then 59 °C for 30 s (primer extension)] followed by 68 °C for 70 s, 68 °C for 2 min and then hold at 4 °C.

Families 3-5. *ENAM* Intron 9: NG_013024.1:g.14101delG c.588+1delG; NM_031889.2: c.588+1delG; p.(Arg179_Gly196)
9F: TGGTAAGGAGGATTGCCAAC
9R ATCTTTGGACCACTGCGTTG; RC: CAAGCCAGTGGTCCAAAGAT
Primers generate a 504 bp amplification product.
PCR conditions: template denaturation @ 94 °C for 2 min, then [35 cycles of 94 °C for 15 s (primer annealing) then 59 °C for 30 s (primer extension)] followed by 68 °C for 30 s, 68 °C for 2 min and then hold at 4 °C.

Family 3. *LAMA3* NG_007853.2:g.99516G>A; NG_007853.2:c.1559G>A; p.(Cys520Tyr)
F: AATTTCCCTTCAACGCAGTG
R: GAAGTGGTGGCAGGTTCCTA; RC: TAGGAACCTGCCACCACTTC
Primers generate a 599 bp amplification product.
PCR conditions: template denaturation @ 94 °C for 2 min, then [35 cycles of 94 °C for 15 s (primer annealing) then 59 °C for 30 s (primer extension)] followed by 68 °C for 40 s, 68 °C for 2 min and then hold at 4 °C.

Table S1. PCR primers and amplification conditions. Each PCR reaction contained 5 µL of 10x High Fidelity (HF) PCR buffer, 2 µL of $MgSO_4$, 1 µL of 10 mM dNTP mix, 2 µL of 10 µM Primer mix, 2µL of DNA template, 0.2 µL of Platinum Taq DNA Polymerase HF (5 U/µL) (Invitrogen, Carlsbad, CA, USA) and raised to 50 µL with distilled water. The reactions were run using a GeneAmp PCR System 9700 (Applied Biosystems, Foster City, CA, USA) Thermocycler.

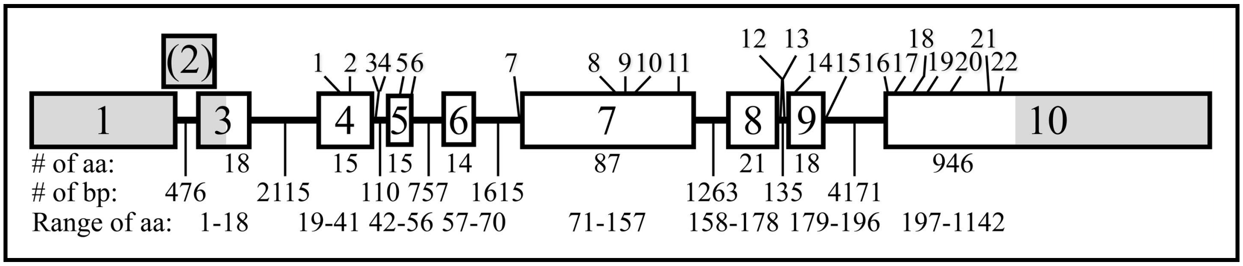

ENAM Disease-Causing Mutations

#	Location	NG_013024.1	NM_031889.2	Protein	References
1	Exon 4	g.7964T>G	c.92T>G	p.(Leu31Arg)[a]	1
2	Exon 4	g.7979delA	c.107delA	p.(Asn36Ilefs*22)[b]	2
3	Intron 4	g.7996G>A	c.123+1G>A	p.(Val19-Pro41del)[c]	3
4	Intron 4	g.7997T>G	c.123+2T>G	p.(Val19-Pro41del)[c]	4
5	Exon 5	g.8121delA	c.139delA	p.(Met47Cysfs*11)[d]	5
6	Exon 5	g.8139A>T	c.157A>T	p.(Lys53*)	6,7
7	Intron 6	g.10563A>C	c.211-2A>C	p.Met71-Gln157del[e]	8
8	Exon 7	g.10712C>T	c.358C>T	p.(Gln120*)	9
9	Exon 7	g.10749dupA	c.395dupA	p.(Pro133Alafs*13)[f]	F1
10	Exon 7	g.10760_10761insTCAAAAAAGCCGACCACAA	c.406_407insTCAAAAAAGCCGACCACAA	p.(Lys136Ilefs*16)[g]	5
11	Exon 7	g.10808G>T	c.454G>T	p.(Glu152*)	9
12	Intron 8	g.12152G>A	c.534+1G>A	p.Ala158-Gln178del[h]	10-12
13	Intron 8	g.14045A>G	c.535-2A>G	p.(Arg179-Gly196del)[i]	13
14	Exon 9	g.14048G>T	c.536G>T	p.(Arg179Met)[j]	14
15	Intron 9	g.14101delG	c.588+1delG	p.Asn197Ilefs*81[k]	F3-F5 8,13,15-18
16	Exon 10	g.18330C>T	c.647C>T	p.(Ser216Leu)[l]	19
17	Exon 10	g18420C>A	c.737C>A	p.(Ser246*)	20
18	Exon 10	g.18704_18705insGTCAGTACCAGTACTGTGTCA	c.1021_1022insGTCAGTACCAGTACTGTGTCA	p.(Val340_Met341insSerGlnTyrGlnTyrCysVal)	20
19	Exon 10	g.18942_18943insAG	c.1259_1260insAG	p.(Pro422Valfs*27)[m]	F2 13,16,17,20-23
20	Exon 10	g.19525C>G	c.1842C>G	p.(Tyr614*)	4
21	Exon 10	g.20446delT	c.2763delT	p.(Asp921Glufs*32)[n]	F2
22	Exon 10	g.20674delT	c.2991delT	p.(Leu998Trpfs*65)[o]	21

Table S2. Known AI-causing *ENAM* mutations. *ENAM* gene diagram showing the positions of all currently published disease-causing mutations (top). Exons are numbered boxes; introns are lines connecting the exons. Shaded exon regions are non-coding. Mutation sites are numbered above. Exon (2) is homologous to a mouse exon in this position, but is not included in the human *ENAM* cDNA reference sequence [24]. It is included here to keep the exon numbers consistent with precedent and previously publications [16]. ***Key:*** aa, amino acids; bp, basepairs. Table of all reported AI-causing mutations in *ENAM* (bottom). The gene numbers start from the first nucleotide of the *ENAM* genomic reference sequence (NG_013024.1). The cDNA numbers start from the first nucleotide of the *ENAM* translation initiation site (NM_031889.2).

Notes for Table I

a. Amino acid substitution in signal peptide (Met1-Ala39).
b. Adds ILLLCQCTCPECLDLAVKVRR* after Gly35.
c. Assumes the skipping of Exon 3, affecting the signal peptide.
d. Adds CLDLAVKVRR* after Arg46.
e. Assumes the skipping of Exon 6.
f. Adds ATTKAAFEAAIT* after Lys132.
g. Adds IKKADHKAAFEAAIT* after Gln135; error in LOVE listing.
h. Assumes the skipping of Exon 7.
i. Assumes the skipping of Exon 8; originally reported as c.816A>C, which erred by counting from the beginning of the cDNA rather than the ATG. (Wright 2011).
j. The possibility that this mutation did not cause the AI in this family was raised during an analysis showing a lack of conservation of Arg179 in mammalian *Enam* sequences [25]. Additional doubt is raised by the description that the "affected teeth of these patients had a distinctive honey-yellowish colour" (which is not consistent with the appearance of other AI-causing *ENAM* mutations) and by the poor quality of the published DNA sequencing chromatogram, which appears to have been too close to the primer to read the sequence reliably [14]. The SIFT score was 0.084 (tolerated); Polyphen HDIV was 0.998 (probably damaging); Polyphen HVAR was 0.911 (probably damaging).
k. Assumes that splicing occurs normally using the last G of Exon 8 to complete the GT splicing junction causing a frameshift by the loss of one nucleotide from the end of Exon 8. This frameshift would add the following 80 amino acids after Gly196: ILTLDILDIMALGVALLIIQKKCLNKILKNPKKKILLKQK VQAQNPQLIQQSLRRILPNQILKGVREEMTPAPQETVPQD*
l. Removes a Golgi Casein Kinase phosphorylation site.
m. Adds VPNLALLFAMKKSKIQRRSPWVQKNK* after Gly421.
n. Adds ESSPQQASYQAKETAQRRGKAKTLLEMMCPR* after Arg920.
o. Adds WNKFLKTTSSMKELLTLLLSSLLLVHLMKAPIQKASKVKSKKMRVR GSKDHLTFCICHALAPN* after Ile997.

Table S2 References

1. Brookes SJ, Barron MJ, Smith CEL, et al. Amelogenesis imperfecta caused by N-terminal enamelin point mutations in mice and men is driven by endoplasmic reticulum stress. *Hum Mol Genet.* 2017;26(10):1863-1876.
2. Simmer SG, Estrella NM, Milkovich RN, Hu JC. Autosomal dominant amelogenesis imperfecta associated with ENAM frameshift mutation p.Asn36Ilefs56. *Clin Genet.* 2013;83(2):195-197.
3. Prasad MK, Geoffroy V, Vicaire S, et al. A targeted next-generation sequencing assay for the molecular diagnosis of genetic disorders with orodental involvement. *J Med Genet.* 2016;53(2):98-110.
4. Koruyucu M, Kang J, Kim YJ, et al. Hypoplastic AI with Highly Variable Expressivity Caused by ENAM Mutations. *J Dent Res.* 2018;97(9):1064-1069.
5. Wang X, Zhao Y, Yang Y, Qin M. Novel ENAM and LAMB3 mutations in Chinese families with hypoplastic amelogenesis imperfecta. *PLoS One.* 2015;10(3):e0116514.
6. Mardh CK, Backman B, Holmgren G, Hu JC, Simmer JP, Forsman-Semb K. A nonsense mutation in the enamelin gene causes local hypoplastic autosomal dominant amelogenesis imperfecta (AIH2). *Hum Mol Genet.* 2002;11(9):1069-1074.
7. Kim JW, Simmer JP, Lin BP, Seymen F, Bartlett JD, Hu JC. Mutational analysis of candidate genes in 24 amelogenesis imperfecta families. *Eur J Oral Sci.* 2006;114 Suppl 1:3-12.

8. Kim JW, Seymen F, Lin BP, et al. ENAM mutations in autosomal-dominant amelogenesis imperfecta. *J Dent Res.* 2005;84(3):278-282.
9. Seymen F, Lee KE, Koruyucu M, et al. ENAM mutations with incomplete penetrance. *J Dent Res.* 2014;93(10):988-992.
10. Rajpar MH, Harley K, Laing C, Davies RM, Dixon MJ. Mutation of the gene encoding the enamel-specific protein, enamelin, causes autosomal-dominant amelogenesis imperfecta. *Hum Mol Genet.* 2001;10(16):1673-1677.
11. Urzua OB, Ortega PA, Rodriguez ML, Morales BI. Genetic, clinical and molecular analysis of a family affected by amelogenesis imperfecta. *Rev Med Chil.* 2005;133(11):1331-1340.
12. Song YL, Wang CN, Zhang CZ, Yang K, Bian Z. Molecular characterization of amelogenesis imperfecta in Chinese patients. *Cells Tissues Organs.* 2012;196(3):271-279.
13. Wright JT, Torain M, Long K, et al. Amelogenesis imperfecta: genotype-phenotype studies in 71 families. *Cells Tissues Organs.* 2011;194(2-4):279-283.
14. Gutierrez SJ, Chaves M, Torres DM, Briceno I. Identification of a novel mutation in the enamalin gene in a family with autosomal-dominant amelogenesis imperfecta. *Arch Oral Biol.* 2007;52(5):503-506.
15. Kida M, Ariga T, Shirakawa T, Oguchi H, Sakiyama Y. Autosomal-dominant hypoplastic form of amelogenesis imperfecta caused by an enamelin gene mutation at the exon-intron boundary. *J Dent Res.* 2002;81(11):738-742.
16. Hart PS, Michalec MD, Seow WK, Hart TC, Wright JT. Identification of the enamelin (g.8344delG) mutation in a new kindred and presentation of a standardized ENAM nomenclature. *Arch Oral Biol.* 2003;48(8):589-596.
17. Pavlic A, Petelin M, Battelino T. Phenotype and enamel ultrastructure characteristics in patients with ENAM gene mutations g.13185-13186insAG and 8344delG. *Arch Oral Biol.* 2007;52(3):209-217.
18. Pavlic A, Battelino T, Trebusak Podkrajsek K, Ovsenik M. Craniofacial characteristics and genotypes of amelogenesis imperfecta patients. *Eur J Orthod.* 2011;33(3):325-331.
19. Chan HC, Estrella NM, Milkovich RN, Kim JW, Simmer JP, Hu JC. Target gene analyses of 39 amelogenesis imperfecta kindreds. *Eur J Oral Sci.* 2011;119 Suppl 1:311-323.
20. Ozdemir D, Hart PS, Firatli E, Aren G, Ryu OH, Hart TC. Phenotype of ENAM Mutations is Dosage-dependent. *J Dent Res.* 2005;84(11):1036-1041.
21. Kang HY, Seymen F, Lee SK, et al. Candidate gene strategy reveals ENAM mutations. *J Dent Res.* 2009;88(3):266-269.
22. Chan HC, Mai L, Oikonomopoulou A, et al. Altered enamelin phosphorylation site causes amelogenesis imperfecta. *J Dent Res.* 2010;89(7):695-699.
23. Lindemeyer RG, Gibson C, Wright TJ. Amelogenesis imperfecta due to a mutation of the enamelin gene: clinical case. *Pediatr Dent.* 2010;32(1):56-60.
24. Hu JC, Zhang CH, Yang Y, Karrman-Mardh C, Forsman-Semb K, Simmer JP. Cloning and characterization of the mouse and human enamelin genes. *J Dent Res.* 2001;80(3):898-902.
25. Al-Hashimi N, Sire JY, Delgado S. Evolutionary analysis of mammalian enamelin, the largest enamel protein, supports a crucial role for the 32-kDa peptide and reveals selective adaptation in rodents and primates. *J Mol Evol.* 2009;69(6):635-656.

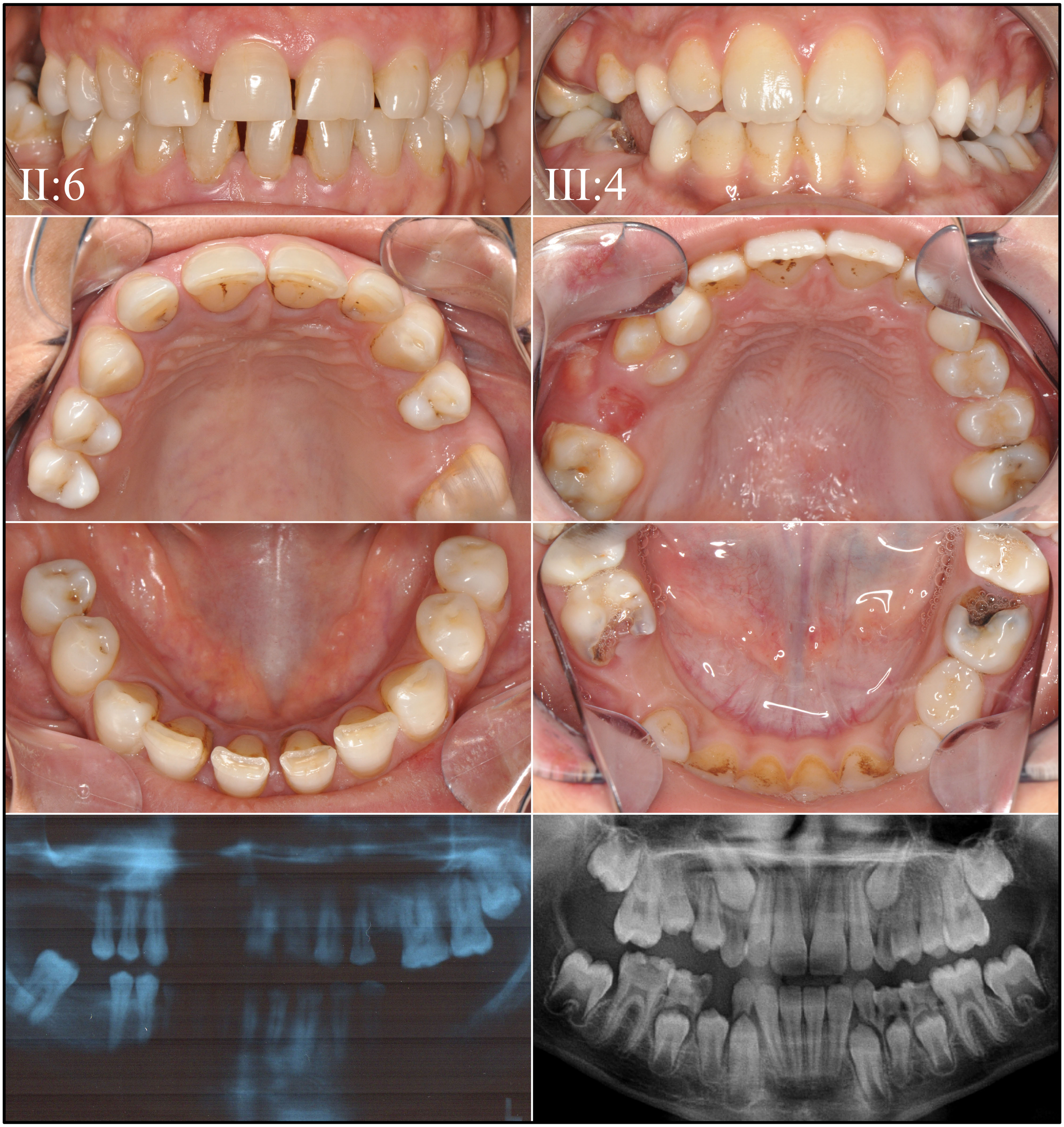

Figure S1. Family 1 unaffected mother (II:6; left) and unaffected younger brother (III:4; right). Both of these persons were negative for the heterozygous *ENAM* mutation that caused the enamel defects in the affected members of the family. It is apparent then that the unrecruited father carried the mutated *ENAM* allele.

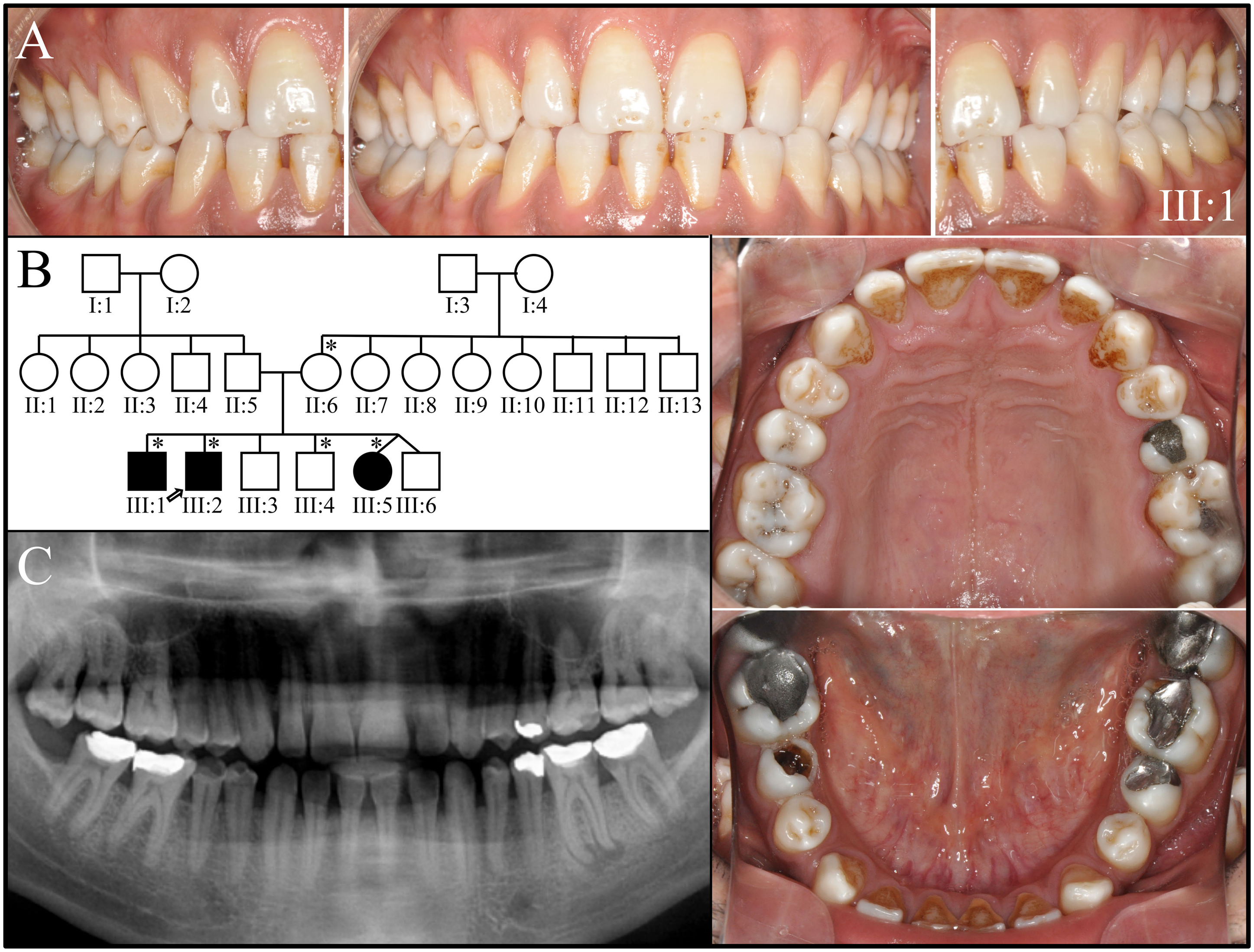

Figure S2. Family 1 affected older brother (III:1). The enamel in the proband's older brother was more mildly affected than his younger siblings. The most prominent feature was well-circumscribed enamel pits and generalized thin enamel evidenced by the spacing among the anterior teeth.

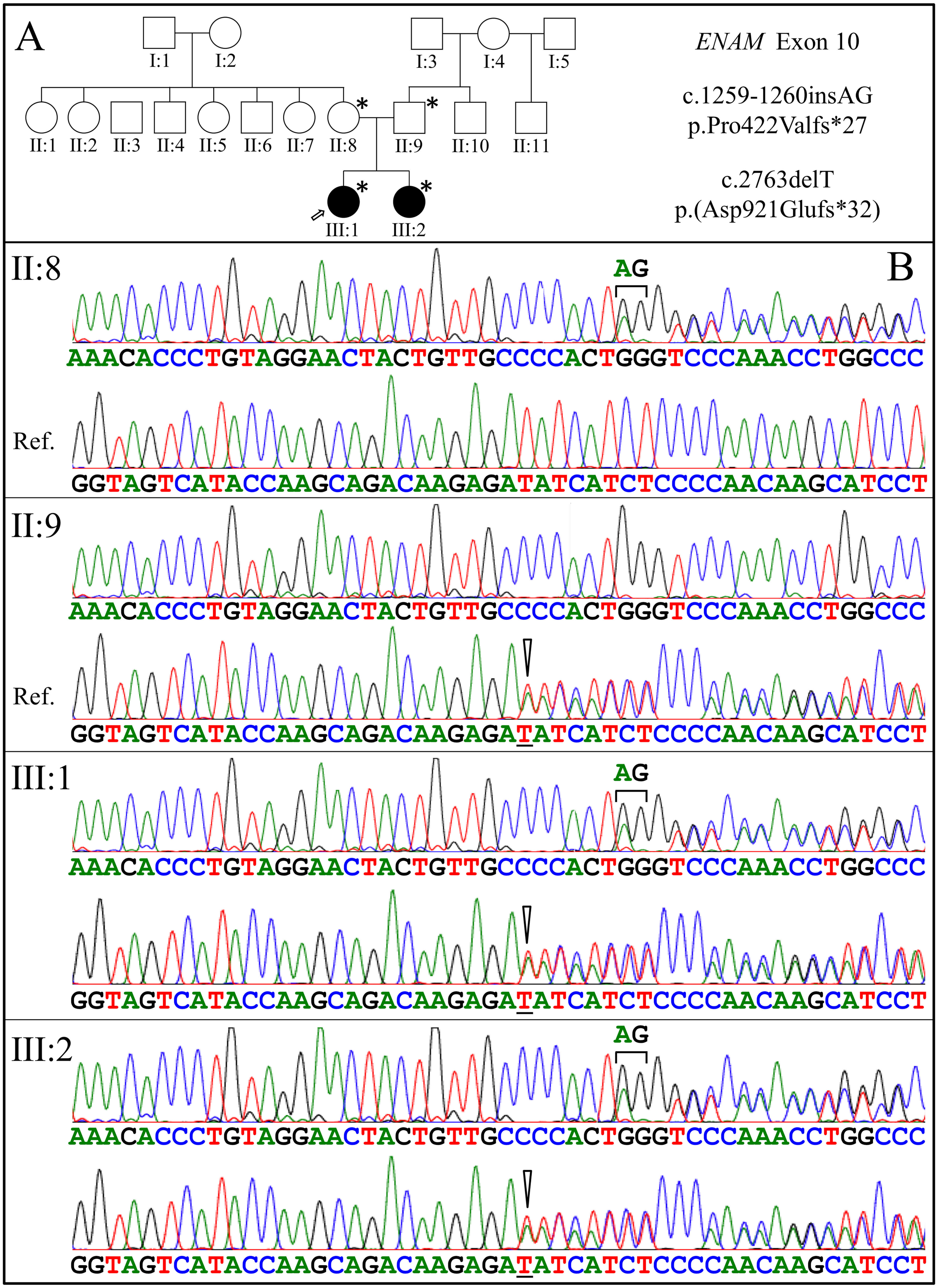

Figure S3. Family 2 pedigree and *ENAM* Exon 10 mutation chromatograms. ***A:*** Pedigree of family with two different *ENAM* mutations exon 10. Asterisks indicate recruited subjects. No photos or radiographs were available. The referring dentist/geneticist detected no enamel phenotype in the parents (II:8 and II:9), who were later found to be heterozygous for one of the two mutations. ***B:*** Sanger sequencing confirmed the distribution of the *ENAM* mutations in the nuclear family.

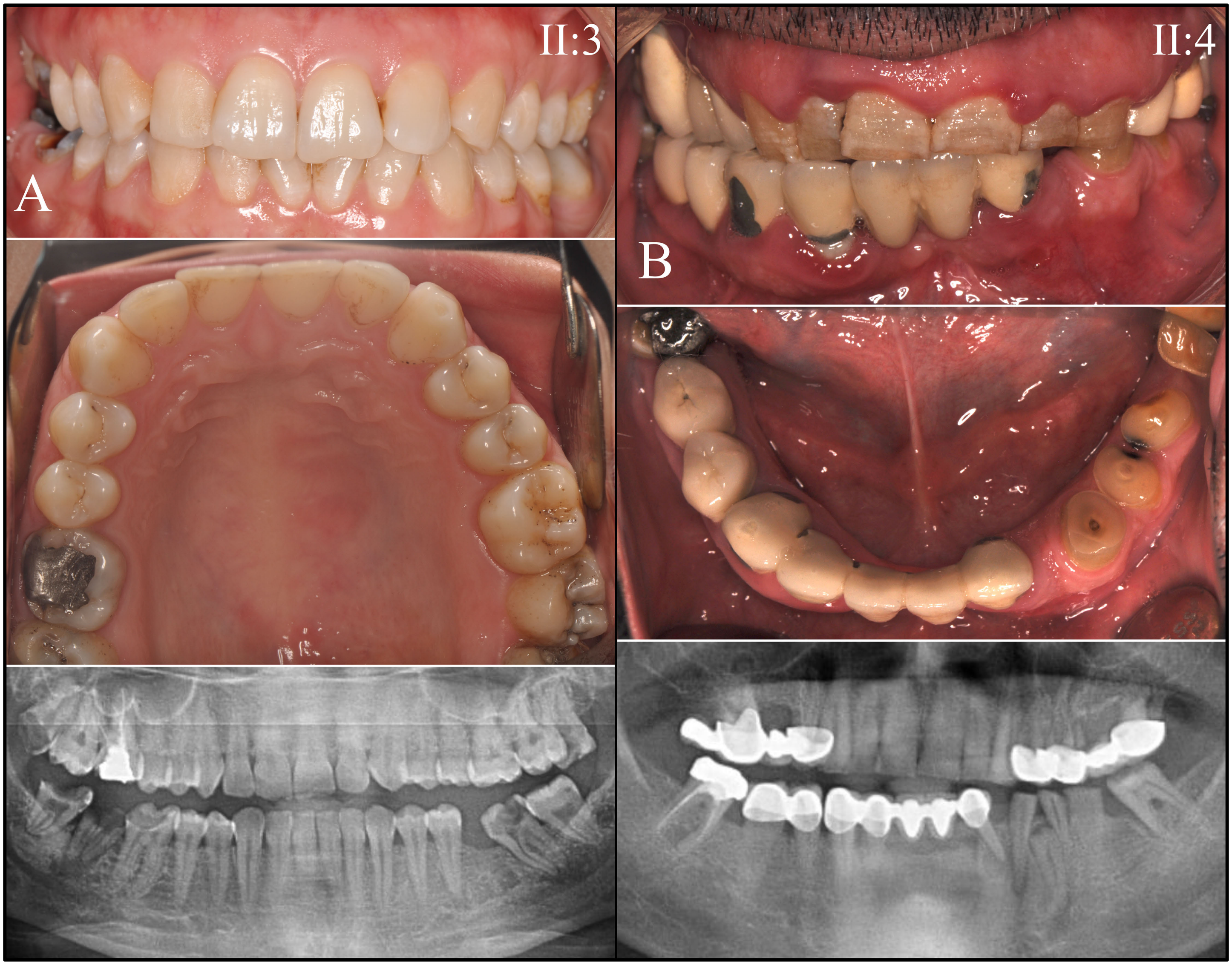

Figure S4. Phenotypes of Family 5 unaffected mother (II:3) and affected father (II:4) of the proband (III:3) who is heterozygous for *ENAM*, c.583delG, p.Asn197Ilefs*81 and *LAMA3*, c.1559G>A, p.Cys520Tyr. All six affected family members (II:2, II:4, III:1, III:2, III:3, III:5) are heterozygous for both mutations, while all of the recruited unaffected family members (II:3, II:5, III:4) have neither mutation.

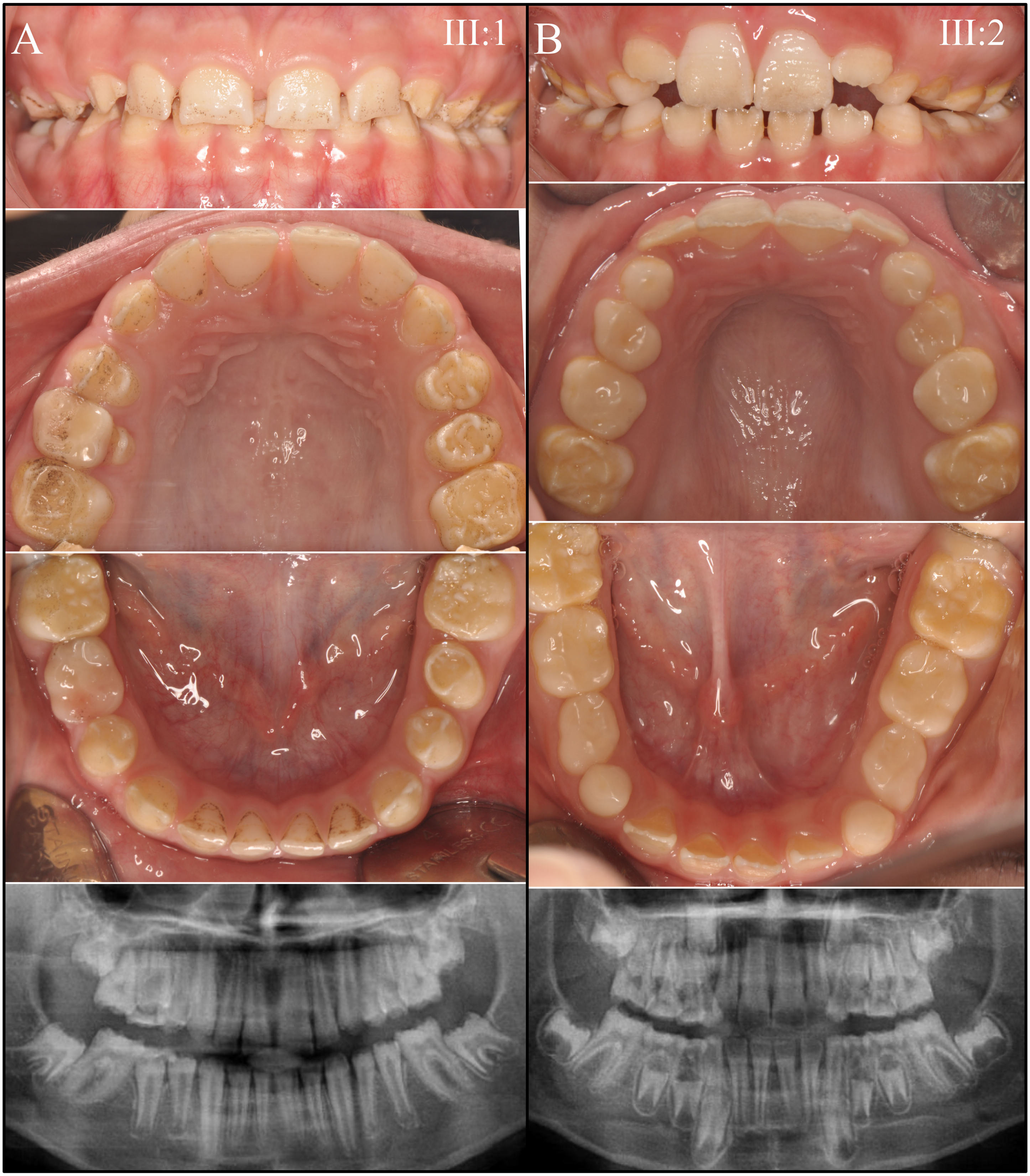

Figure S5. Phenotypes of Family 5 affected cousins: (III:1, female 11y) and (III:2, male 9y). Both of these individuals are heterozygous for the *ENAM*, c.583delG, p.Asn197Ilefs*81 and *LAMA3*, c.1559G>A, p.Cys520Tyr mutations. Note the rough and pitted enamel surface of the maxillary teeth in A as well as the fractured, rough anterior incisal edges in B. The posterior dentition of both individuals showed roughened, irregular occlusal surfaces.

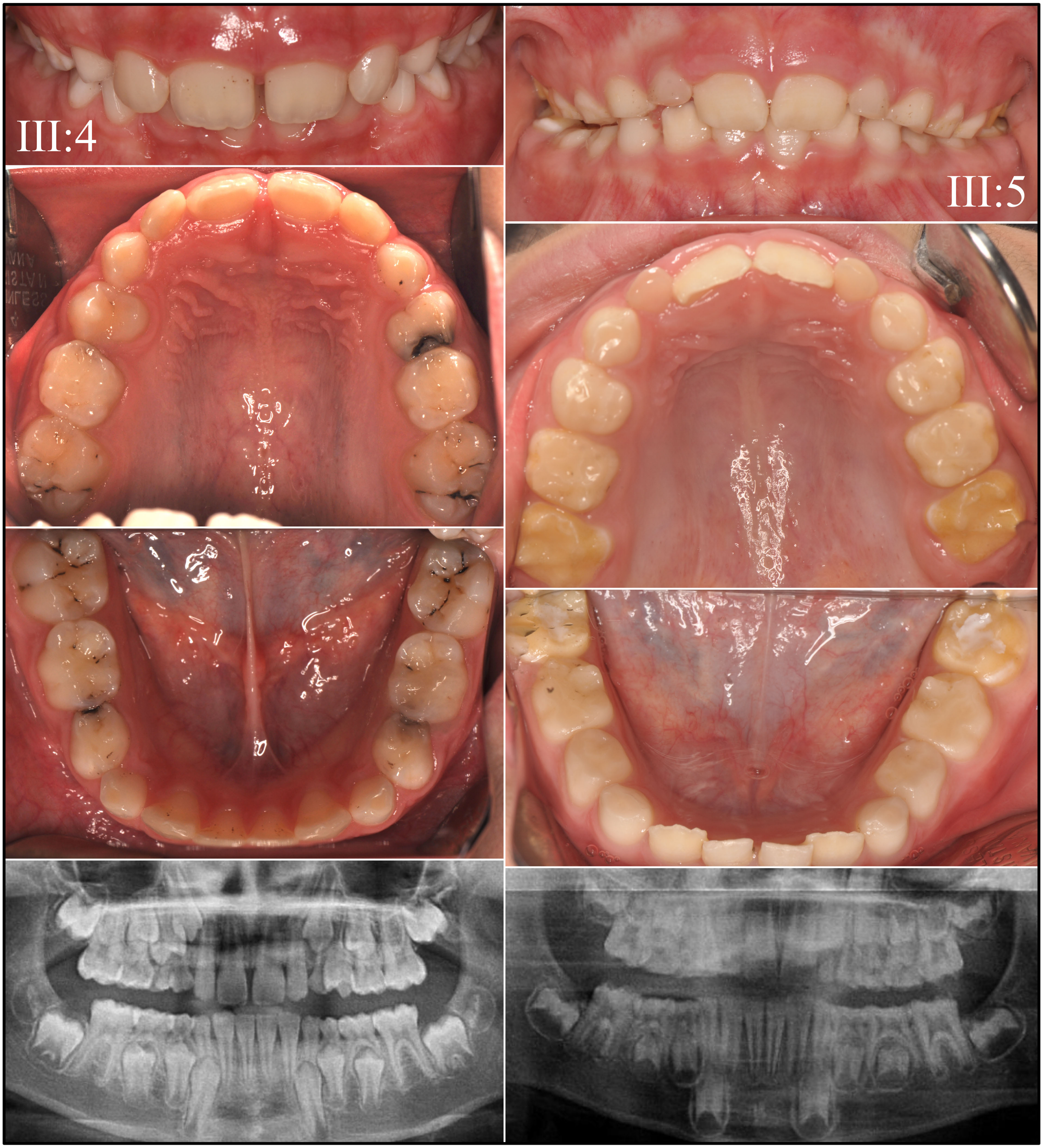

Figure S6. Clinical phenotypes of Family 5 younger siblings (III:4, female 11y and III:5, male 8y). The unaffected younger sister (III:4) does not have a mutation in either *ENAM* or *LAMA3*, while the younger affected brother (III:5) is heterozygous for the *ENAM*, c.588+1delG, p.Asn197Ilefs*81 and *LAMA3*, c.1559G>A/p.Cys520Tyr mutations. Note the fractured, irregular incisal surfaces of the mandibular anterior teeth in (III:5).

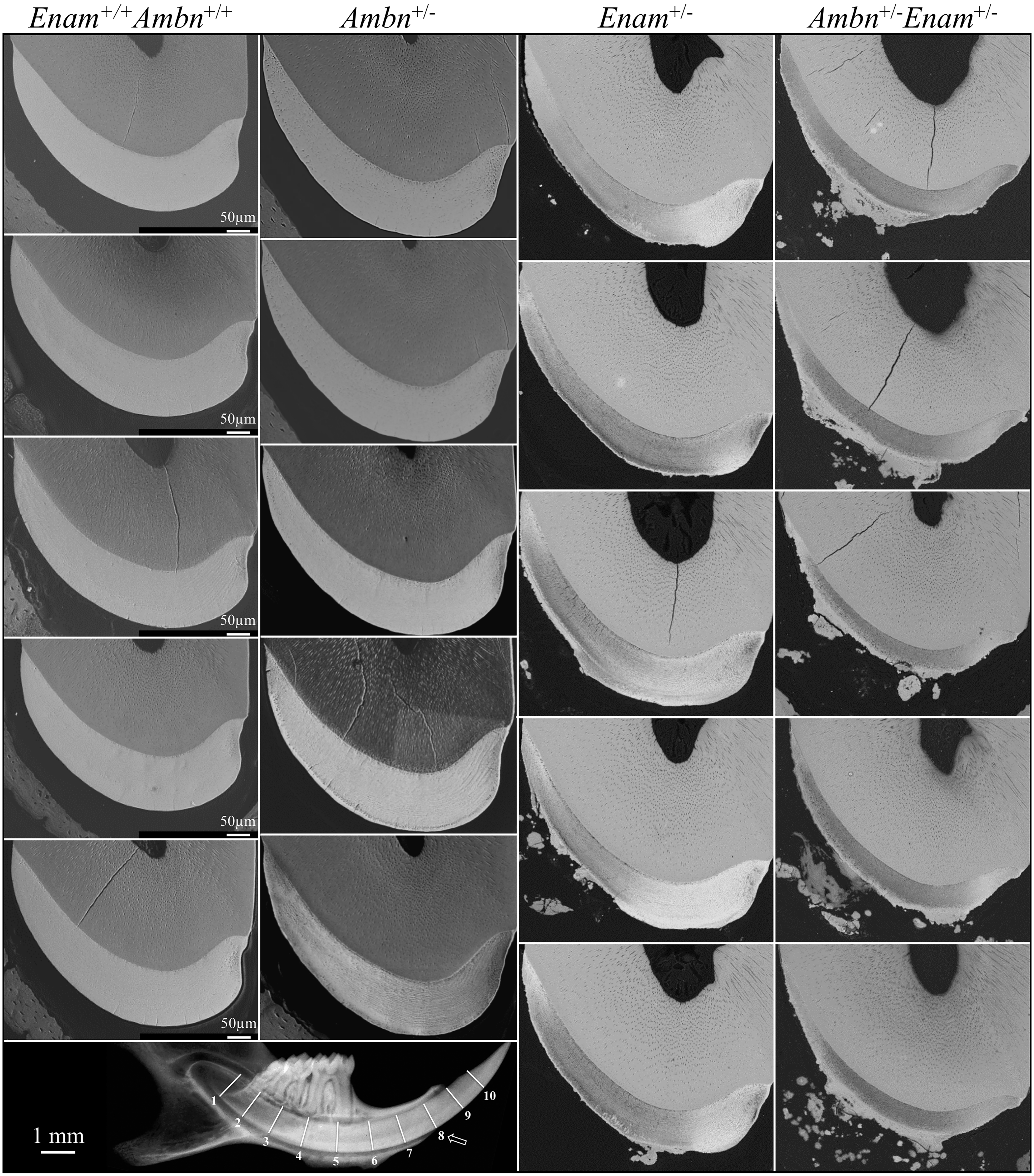

Figure S7. Level 8 cross-sections from 5 incisors from 7-week *Enam*$^{+/+}$*Ambn*$^{+/+}$, *Enam*$^{+/-}$, and *Enam*$^{+/-}$*Ambn*$^{+/-}$ mice. The labial alveolar crest is a standard location to sample incisors to assess essentially completely formed enamel before it has erupted in the oral cavity and potentially altered. Note that the enamel malformations are consistently most severe in the *Enam*$^{+/-}$*Ambn*$^{+/-}$ double heterozygous incisors.

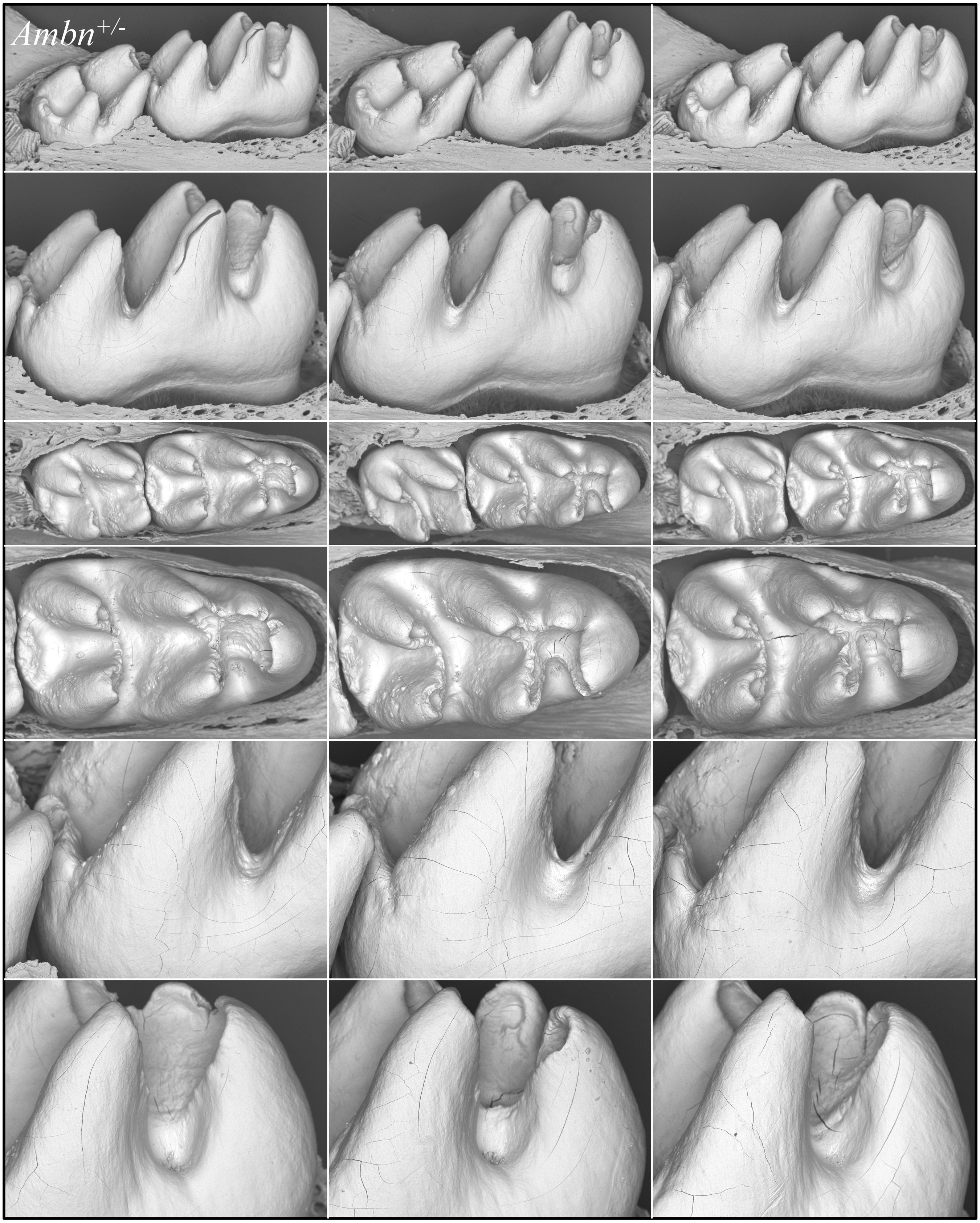

Figure S8. D14 bSEM images of mandibular molars from three *Ambn*$^{+/-}$ mice. Day 14 immediately precedes eruption of the first molars into the oral cavity and is chosen to assess completely formed enamel before it can potentially be altered in the oral cavity. Crown form is normal. There is some minor surface roughness.

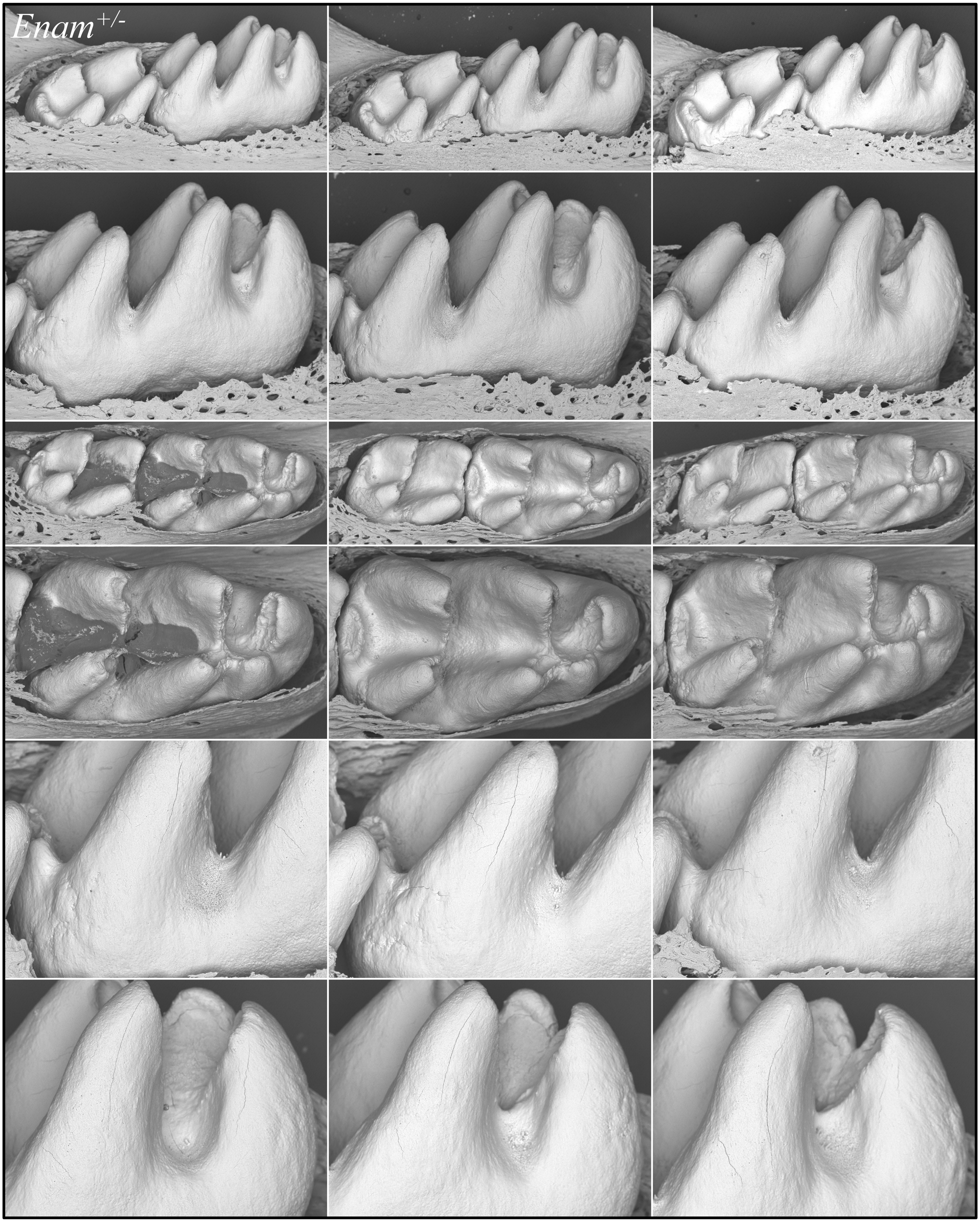

Figure S9. D14 bSEM images of mandibular molars from three *Enam*$^{+/-}$ mice. Crown form is normal. There is minor surface roughness, that is more pronounced than that observed in the *Ambn*$^{+/-}$ molars. The dark gray areas on the occlusal surface of the molars on the left was due to retained soft tissue during preparation.

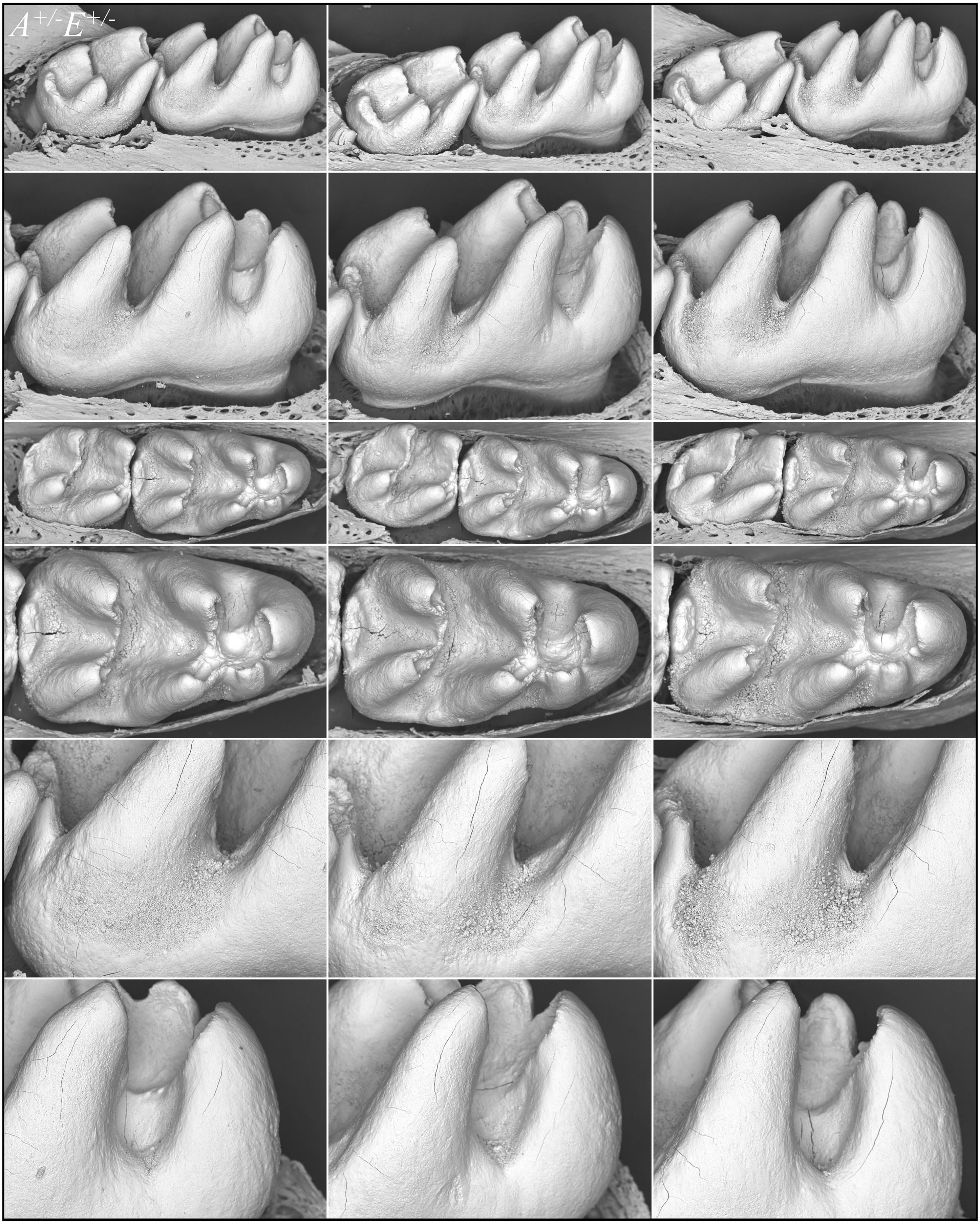

Figure S10. D14 bSEM images of mandibular molars from three *Ambn*$^{+/-}$*Enam*$^{+/-}$ mice. Crown form is essentially normal. There is major surface roughness with extensive regions with surface nodules.
